# Foodborne Illness, Australia, Circa 2000 and Circa 2010

**DOI:** 10.3201/eid2011.131315

**Published:** 2014-11

**Authors:** Martyn Kirk, Laura Ford, Kathryn Glass, Gillian Hall

**Affiliations:** Australian National University, Canberra, Australian Capital Territory, Australia

**Keywords:** foodborne illness, foodborne disease, gastroenteritis, epidemiology, estimate, incidence, hospitalization, death, norovirus, salmonella, campylobacter, toxin, bacteria, parasites, viruses, Australia

## Abstract

Foodborne disease is a major public health problem worldwide. To examine changes in foodborne illness in Australia, we estimated the incidence, hospitalizations, and deaths attributed to contaminated food circa 2010 and recalculated estimates from circa 2000. Approximately 25% of gastroenteritis cases were caused by contaminated food; to account for uncertainty we used simulation techniques to estimate 90% credible intervals. We estimate that circa 2010, 4.1 million foodborne gastroenteritis cases occurred, and circa 2000, 4.3 million cases occurred. Circa 2010, contaminated food was estimated to be responsible for 30,840 gastroenteritis-associated hospitalizations, 76 associated deaths, and 5,140 nongastrointestinal illnesses. Cases of salmonellosis and campylobacteriosis increased from 2000 to 2010 and were the leading causes of gastroenteritis-associated hospitalizations; *Listeria monocytogenes* and nontyphoidal *Salmonella* spp. infections were the leading causes of death. Although the overall incidence of foodborne illnesses declined over time in Australia, cases of foodborne gastroenteritis are still common.

## Abstract 2

Overall incidence of foodborne gastroenteritis declined but remains high, and the incidence of salmonellosis and campylobacteriosis increased.

Foodborne illness is a major public health problem and a common cause of illness and death worldwide. Outbreaks linked to contaminated food can affect the public’s trust and financially harm implicated businesses and associated food industries. Estimates of the effects of foodborne illnesses and individual pathogens provide evidence for policy interventions and food safety regulation. In addition, estimates of changes in the incidence of foodborne illnesses and hospitalizations over time provide information on the effectiveness of changes to food safety standards and regulation.

Many agents can cause foodborne illness; some of these agents are transmitted to humans by other routes as well as by food. Most foodborne illnesses manifest as gastroenteritis, but other presentations, such as meningitis and hepatitis may also result from infection, and sequelae may occur weeks after the acute infection.

Many countries have estimated the incidence of foodborne diseases (*1*–*5*). In Australia in 2000, foodborne incidence, hospitalizations, and deaths were estimated to cost 1.25 billion Australian dollars annually (*6*,*7*). However, since 2000, surveillance has substantially improved, data availability has increased, and methods have been refined. To inform current public health decisions and policies in Australia, we used new methods and datasets to estimate the incidence of infectious gastroenteritis and associated hospitalizations and deaths in Australia circa 2010. We then applied these refined methods to circa 2000 data so that estimates from the 2 periods could be directly compared.

## Methods

We estimated the incidence of illness and the number of hospitalizations and deaths associated with 23 potentially foodborne pathogens or agents in Australia circa 2010 (Technical Appendix 1 Table 1). Pathogens we did not consider relevant were those acquired only overseas (e.g., *Vibrio cholerae, Trichinella spiralis)* and those that cause gastroenteritis but are not proven agents of foodborne disease (e.g., *Clostridium difficile*). Estimates of chronic sequelae from foodborne illnesses are discussed elsewhere in this issue (*8*).

When possible, data for the circa 2010 study period covered 2006–2010, and all denominator data were based on the Australian population during that period (*9*). Estimates of incidence relied on data obtained from 4 sources: notifiable disease surveillance at the national and state levels; outbreak surveillance through the OzFoodNet Outbreak Register; the National Gastroenteritis Survey II (NGSII; http://www.ozfoodnet.gov.au/), a cross-sectional survey; and the Water Quality Study (WQS), a randomized controlled trial (conducted during 1997–1999) of household water treatment to prevent gastroenteritis (*10*,*11*). Estimates of severe illness were determined by using hospitalization and death data. This study was approved by the Australian National University Human Research Ethics Committee. Further details of the data sources and methods are in Technical Appendix 1.

To estimate incidence, hospitalizations, and deaths, we built on our previous methods (*7*), making them similar to those used in the United States (*2*,*3*). We calculated estimates by using simulation techniques in @Risk version 6 (http://www.palisade.com/) with multiple inputs, each with different levels of uncertainty. We used empirical, lognormal, and PERT (program evaluation review technique) probability distributions to model uncertainty in source data and multipliers. Estimates are expressed as probability distributions summarized by a median point estimate with a 90% credible interval (CrI) (Technical Appendix 2).

### Incidence Circa 2010

To estimate the annual incidence of infectious gastroenteritis in Australia circa 2010, we used symptoms included in the NGSII telephone survey conducted during February 2008–January 2009. Case definition has a considerable effect when determining the incidence of gastroenteritis (*12*). To enable a valid comparison of circa 2000 and circa 2010 gastroenteritis estimates, we used the case definition from the earlier study (*13*,*14*). In NGSII, persons were considered case-patients if they had >3 episodes of diarrhea or >2 episodes of vomiting within a 24-h period during the preceding 4 weeks and did not report a noninfectious cause for their illness. However, for persons who had concomitant respiratory symptoms, we applied a stricter definition: >4 episodes of diarrhea and/or >3 episodes of vomiting (*15*). In NGSII, 4.5% (341/7,578) of survey respondents reported gastroenteritis in the preceding 4 weeks, equating to 0.74 gastroenteritis episodes per person per year (95% CI 0.64–0.84) or 15.9 million cases annually in Australia.

We used 2 main approaches to estimate the incidence of foodborne illness caused by specific pathogens or illnesses. Our preferred approach was the surveillance approach, in which we estimated the community incidence of illness by applying an underreporting multiplier to scale up data from notifiable disease surveillance. When these data were not available, we used a pathogen fraction approach, in which we estimated the percentage of overall gastroenteritis caused by specific pathogens. When data were unavailable by either of these approaches, we used other surveillance data, such as outbreak data. Approach-specific flow charts are provided in Technical Appendix 2.

Using the surveillance approach, we adjusted for underreporting of community cases to public health surveillance. We used findings from an underreporting multiplier study in Australia (*16*) for moderate illnesses and bloody diarrhea. For serious illnesses, we assumed the underreporting factor as 1 illness reported for every 2 that occurred in the community, as used by Mead et al. (*17*) and Scallan et al. (*2*). We applied another multiplier to outbreak surveillance data to adjust for underreporting when only outbreak cases were notified (Technical Appendix 2).

When we used the pathogen fraction approach, our main data source was the WQS (*10*,*11*). The WQS provided data on the proportion of gastroenteritis episodes caused by specific pathogens, and we applied those proportions to total foodborne illness incidence data from the NGSII. However, the WQS was conducted before rotavirus vaccine was added to the Australian vaccination schedule. To account for the effect of the vaccine on infection incidence, we calculated a time-trend multiplier by using age-specific hospitalization data from before and after introduction of rotavirus vaccine (*18*).

We used the surveillance approach for cases caused by 16 pathogens, of which 11 were from the National Notifiable Diseases Surveillance System (NNDSS; http://www.health.gov.au/internet/main/publishing.nsf/Content/cda-surveil-nndss-nndssintro.htm) and 5 were from outbreak data. We used the pathogen fraction approach for cases caused by 6 pathogens. In addition, because local data were lacking, we applied US seroprevalence data to the Australian population data to estimate the incidence of toxoplasmosis (Technical Appendix 2).

### Incidence Circa 2000

Methods for calculating incidence have changed since the circa 2000 estimates were determined (*7*); the changes include updated underreporting multipliers (*16*), more rigorous expert elicitation (*19*), and new estimates of the foodborne multipliers for some pathogens. These changes could result in a potentially misleading comparison of circa 2010 and circa 2000 findings. We recalculated estimates for circa 2000 by using the original data with methods identical to those used for circa 2010 data. Updated estimates of the total incidence of foodborne gastroenteritis were determined by using the original 2001 National Gastroenteritis Survey, together with the 2010 foodborne proportion of 25% (compared with 32% in the circa 2000 study). To recalculate the circa 2000 estimates, we replaced multipliers used in that study with circa 2010 multipliers and applied them to 1996–2000 data from NNDSS for *Campylobacter* spp., nontyphoidal *Salmonella enterica* serotypes (hereafter referred to as nontyphoidal *Salmonella* spp.), *S. enterica* serotype Typhi, *Shigella* spp., hepatitis A virus, and *Listeria monocytogenes* infections and to 1996–2000 surveillance data from the state of Victoria, Australia, for *Giardia lamblia*. Only pathogens for which we had surveillance data from both periods were included in this analysis.

### Hospitalizations and Deaths

We estimated the annual number of hospitalizations for foodborne illnesses by using 2006–2010 state and territory hospitalization data (http://www.aihw.gov.au/hospitals/australian-hospital-statistics/) for which principal and additional diagnoses were based on the Australian modification of the 10th International Classification of Diseases (*20*), and we estimated the annual number deaths by using 2001–2010 Australian Bureau of Statistics’ national death data for underlying or contributing cause (http://www.aihw.gov.au/deaths/). Reports for a large number of hospitalizations and deaths caused by gastrointestinal illnesses that were presumed infectious did not identify a specific pathogen.

We adjusted for travel-associated cases and estimated the proportions of foodborne disease–associated hospitalizations and deaths (Technical Appendix 3). Because the recorded hospitalizations and deaths associated with each pathogen reflect only laboratory-confirmed cases, we applied an underdiagnosis multiplier of 2 (range 1–3). This multiplier has been used in other studies (*2*,*7*,*17*) but never validated. Assuming that outbreaks provide a representative denominator population from which to calculate the proportion of hospitalized case-patients, we confirmed the appropriateness of the multiplier by using the OzFoodNet Outbreak Register (http://www.ozfoodnet.gov.au/) to calculate, for a number of pathogens, the proportion of hospitalized case-patients. For the included pathogens, we compared this proportion with the ratio of our estimated yearly hospitalizations to yearly illnesses.

### Domestically Acquired Multiplier

To exclude infections acquired overseas, we applied a domestically acquired multiplier to all pathogens to adjust the total incidence data. For many pathogens, this multiplier was estimated from surveillance data from states and territories that recorded illnesses acquired overseas; variability by state and by year was used to inform uncertainty in the multiplier. Other pathogens causing illness of short duration were assumed to be 100% domestically acquired. Details, by pathogen, are provided in Technical Appendix 4.

### Proportion Foodborne Multiplier

To estimate the total number of foodborne infections caused by each pathogen, we applied a pathogen-specific proportion foodborne multiplier to all pathogens (Technical Appendix 2 Table 2). The proportion foodborne multiplier was estimated for 9 pathogens in 2009 by using an expert elicitation process (*19*), and the multipliers for another 9 pathogens were estimated by using a similar expert elicitation study in 2005 (*21*). All illnesses due to seafood toxins were assumed to be caused by food, and multipliers for 3 viruses were assumed to be equal to those for similar pathogens.

The estimated annual number of gastroenteritis cases caused by 18 known pathogens/parasites for the circa 2010 study period is listed in Table 1. An estimated 25% of the cases were caused by contaminated food, of which 36%, 16%, and 11% were caused by bacteria, viruses, and parasites, respectively. Given an absence of other data sources, we applied this overall foodborne proportion of 25% to the total number of gastroenteritis cases to determine the number caused by contaminated food (*3*,*17*,*22*).

**Table 1:** Estimated number of gastroenteritis cases caused by domestically acquired pathogens, Australia, circa 2010*

*Causative agent*	*Total no. cases, median (90% CrI)*	*% Cases caused by contaminated food, median (90% CrI)*	*No. cases caused by contaminated food, median (90% CrI)*
Bacterium			
* Bacillus cereus*	3,350 (900–10,100)	100 (98–100)	3,350 (900–10,100)
*Campylobacter* spp.	234,000 (147,000–374,000)	77 (62–89)	179,000 (108,500–290,000)
* Clostridium perfringens*	16,500 (2,600–53,400)	98 (86–100)	16,100 (2,550–50,600)
STEC	4,300 (2,050–9,500)	56 (32–83)	2,350 (950–5,850)
Other pathogenic *E. coli*	1,100,000 (833,000–1,450,000)	23 (8–55)	255,000 (85,800–632,000)
*Salmonella* spp, nontyphoidal	56,200 (31,900–101,000)	72 (53–86)	39,600 (21,200–73,400)
*Salmonella enterica* ser. Typhi	20 (8–45)	75 (2–97)	15 (5–30)
*Shigella* spp.	3,000 (1,650–5,400)	12 (5–23)	350 (150–850)
* Staphylococcus aureus*	1,300 (200–7,050)	100 (95–100)	1,300 (200–7,000)
* Vibrio parahaemolyticus*	60 (15–170)	75 (5–96)	40 (10–120)
* Yersinia enterocolitica*	1,500 (900–2,500)	84 (28–94)	1,150 (650–1,950)
Virus			
Adenovirus	88,400 (28,800–205,000)	2 (1–3)	1,650 (500–4,650)
Astrovirus	67,100 (20,900–155,000)	2 (1–3)	1,300 (350–3,400)
Norovirus	1,550,000 (1,220,000–1,940,000)	18 (5–35)	276,000 (78,100–563,000)
Rotavirus	44,800 (18,500–90,800)	2 (1–3)	850 (300–2,000)
Sapovirus	81,600 (63,400–102,000)	18 (5–35)	15,000 (7,450–24,300)
Parasite			
*Cryptosporidium* spp.	17,900 (8,150–39,800)	10 (1–27)	1,700 (150–6,100)
* Giardia lamblia*	32,800 (19,800–56,400)	6 (1–50)	3,700 (800–10,600)
Subtotal	3,090,000 (2,810,000–3,900,000)	25 (13–42)	798,000 (528,000–1,310,000)
Unknown etiology	12,800,000 (10,500,000–14,500,000)	25 (13–42)	3,310,000 (1,800,000–5,152,000)
Total	15,900,000 (13,700,000–18,000,000)	25 (13–42)	4,110,000 (2,330,000–6,390,000)

*All estimates were based on an empirical distribution of the Australian population in the June quarter of 2006–2010; for the parameters of these distributions, see online Technical Appendix 4 (http://wwwnc.cdc.gov/eid/article/20/11/13-1315-Techapp4.pdf). CrI, credible interval; *E. coli*, *Escherichia coli*; STEC, Shiga toxin–producing *E. coli.*

## Results

### Incidence

#### Foodborne Gastroenteritis Circa 2010

We estimated that each year circa 2010, 4.1 million domestically acquired cases (90% CrI 2.3–6.4) of foodborne gastroenteritis occurred in Australia. Of those annual cases, 0.8 million were caused by the 18 pathogens that were known agents of gastroenteritis, and the remaining 3.3 million cases were caused by unknown or unidentified pathogens (Table 1; Technical Appendix 5). Pathogenic *Escherichia coli*, norovirus, *Campylobacter* spp., and nontyphoidal *Salmonella* spp. were the most common causes of foodborne gastroenteritis; together, they were responsible for 93% of the foodborne illnesses caused by known pathogens.

#### Foodborne Nongastrointestinal Illness Circa 2010

In addition to causing foodborne gastroenteritis, contaminated food also caused 5,140 cases (90% CrI 3,530–7,980) of nongastrointestinal illness in Australia circa 2010 (Table 2). Toxoplasmosis was the most common foodborne nongastrointestinal illness; 3,750 cases (90% CrI 1,400–7,150) occurred each year. The percentage of foodborne illnesses caused by nongastroenteric agents ranged from a low of 12% for hepatitis A infection to a high of 100% for scombrotoxicosis and ciguatera.

**Table 2:** Estimated number of acute foodborne illness cases caused by domestically acquired pathogens and agents that do not result in gastroenteritis, Australia, circa 2010*

Illness	% Foodborne, median (90% CrI)	No. illnesses, median (90% CrI)
Hepatitis A virus infection	12 (5–24)	40 (10–100)
Listeriosis	98 (90–100)	150 (50–200)
Toxoplasmosis	31 (4–74)	3,750 (1,400–7,150)
Ciguatera	100 (100–100)	150 (40–300)
Scombrotoxicosis	100 (100–100)	1,050 (0–2,450)
Total	40 (25–59)	5,140 (3,530–7,980)

*All estimates were based on an empirical distribution of the Australian population in the June quarter of 2006–2010; for the parameters of these distributions, see online Technical Appendix 4 (http://wwwnc.cdc.gov/eid/article/20/11/13-1315-Techapp4.pdf). CrI, credible interval.

#### Comparison of Circa 2010 Estimates with Circa 2000 Estimates

When we applied the newer estimation methods, including the new proportion foodborne multiplier (i.e., 25%), to circa 2000 data, the annual number of foodborne gastroenteritis cases was 4.3 million (90% CrI: 2.2–7.3). That total translates to a circa 2000 incidence of 224,000 cases/million population (90% CrI 116,000–374,000). Comparison of the circa 2010 incidence (186,000 cases/million population; 90% CrI 105,000–289,000) with the circa 2000 incidence showed a 17% decreased incidence of foodborne gastroenteritis between 2000 and 2010, although the CrI included 1 (rate ratio [RR] 0.83, 90% CrI 0.4–1.8). Similar recalculation of circa 2000 estimates for key gastrointestinal pathogens showed a total of 28,000 cases (90% CrI 15,000–50,000) of foodborne salmonellosis each year (incidence 1,500 cases/million population, 90% CrI 800–2,700) and 139,000 cases (90% CrI 82,500–227,000) of foodborne campylobacteriosis each year (incidence 7,400 cases/million population, 90% CrI 4,500–12,200) (Table 3). Comparison of the circa 2000 and circa 2010 incidence rates showed RRs of 1.24 (90% CrI 0.5–2.8) for foodborne salmonellosis and 1.13 (90% CrI 0.5–2.3) for foodborne campylobacteriosis, although the CrI included 1. CrIs include uncertainty derived from incidence multipliers and were considerably wider than intervals for ratios derived from raw surveillance data.

**Table 3:** Comparison of estimates of the annual number of cases and incidence rates for foodborne gastroenteritis and key foodborne pathogens, Australia, circa 2000 and circa 2010*

*Foodborne illness/pathogen*	*Circa 2000*			*Circa 2010*		*RR (90% CrI)*
	No. cases, median (90% CrI)	Rate per million population (90% CrI)		No. cases, median 90% (CrI)	Rate per million population (90% CrI)	
Gastroenteritis	4.3 million (2.2–7.3 million)	224,000 (116,000–374,000)		4.1 million (2.3–6.4 million)	186,000 (105,000–289,000)	0.83 (0.4–1.8)
*Campylobacter* spp.	139,000 (82,500–227,000)	7,400 (4,500–12,200)		179,000 (108,500–290,000)	8,400 (5,050–13,650)	1.13 (0.5–2.3)
*Salmonella* spp., nontyphoidal	28,000 (15,000–50,000)	1,500 (800–2,700)		39,600 (21,200–73,400)	1,850 (1,000–3,350)	1.24 (0.5–2.8)
*Salmonella* *enterica* ser. Typhi	9 (3–21)	0.5 (0–1)		15 (5–30)	0.6 (0–1)	1.2 (0.5–2.6)
*Shigella* spp.	515 (175–1,300)	28 (9–70)		350 (150–850)	16 (6–40)	0.57 (0.2–2.3)
Hepatitis A virus	245 (65–725)	13 (3–40)		40 (10–100)	2 (1–5)	0.15 (0.06–0.4)
*Listeria monocytogenes*	125 (70–185)	7 (4–10)		150 (50–100)	7 (3–10)	1 (0.4–1.9)
*Giardia lamblia*	2,600 (565–7,400)	140 (30–405)		3,700 (800–10,600)	175 (35–490)	1.25 (0.5–1.9)

*Estimates are based on an empirical distribution of the Australian population in the June quarter of 1996–2000 (circa 2000 estimates) and 2006–2010 (circa 2010 estimates); for the parameters of these distributions, see online Technical Appendix 4 (http://wwwnc.cdc.gov/eid/article/20/11/13-1315-Techapp4.pdf). CrI, credible interval; RR, rate ratio.

### Hospitalizations

Circa 2010, there were an estimated 30,600 hospitalizations (90% CrI 28,000–34,000) for foodborne gastroenteritis and 240 hospitalizations (90% CrI 180–350) for nongastrointestinal foodborne illnesses (Table 4). Approximately 5,900 of all hospitalizations for gastroenteritis were for illnesses caused by known pathogens, of which *Campylobacter* spp. and nontyphoidal *Salmonella* spp. were the leading causes of hospitalization, and *L. monocytogenes* was the leading cause of nongastrointestinal illnesses requiring hospitalization. The remaining 24,700 hospitalizations were for gastroenteritis of unknown etiology.

**Table 4:** Estimated annual number of hospitalizations and deaths resulting from domestically acquired foodborne pathogens, parasites, and diseases, Australia, circa 2010*

*Illness, causative agent/illness*	*ICD-10-AM code*	No. hospitalizations, median *(90% CrI)*	*No. deaths, median (90% CrI)*
Gastrointestinal illness, cause			
Bacterium			
* Bacillus cereus*	A05.4	25 (4–45)	0
*Campylobacter* spp.	A04.5	3,200 (2,100–4,500)	3 (2–4)
* Clostridium perfringens*	A05.2	0 (0–2)	1 (0–1)
STEC	A04.3	7 (2–15)	0
*O*ther pathogenic *E. coli*	A04.0, A04.1, A04.4	20 (6–50)	0 (0–1)
*Salmonella* spp., nontyphoidal	A02.0-A02.9	2,100 (1,300–3,000)	15 (8–20)
*Salmonella* *enterica* ser. Typhi	A01.0	15 (6–35)	0
*Shigella* spp.	A03	25 (9–50)	0
* Staphylococcus aureus*	A05.0	10 (7–20)	0
* Vibrio parahaemolyticus*	A05.3	1 (0–1)	0
* Yersinia enterocolitica*	A04.6	35 (10–65)	1 (0–1)
Virus			
Adenovirus	A08.2	15 (8–25)	0
Astrovirus	NA	NA	NA
Norovirus	A08.1	150 (35–350)	1 (0–2)
Rotavirus	A08.0	50 (30–100)	0 (0–0)
Sapovirus	NA	NA	NA
Parasite			
*Cryptosporidium* spp.	A07.2	40 (6–100)	0
* Giardia lamblia*	A07.1	100 (25–300)	0
Subtotal		5,900 (4,700–7,500)	21 (14–26)
Unknown etiology	A08.4, A09, A09.0, A09.9	24,700 (22,600–27,800)	39 (27–54)
Total		30,600 (28,000–34,000)	60 (53–63)
Nongastrointestinal illness			
Hepatitis A	B15.9	20 (6–50)	0 (0–2)
Listeriosis	A32	150 (100–250)	15 (9–20)
Toxoplasmosis	B58	30 (10–60)	1 (0–2)
Ciguatera	T61.0	25 (10–40)	0
Scombrotoxicosis	T61.1	8 (5–10)	0
Total		240 (180–350)	16 (10–21)

*All estimates based on an empirical distribution of the Australian population in the June quarter of 2006–2010 for hospitalizations and 2001–2010 for death; see online Technical Appendix 3 (http://wwwnc.cdc.gov/eid/article/20/11/13-1315-Techapp3.pdf) for the methods used to determine these estimates. CrI, credible interval; ICD-10-AM, Australian modification of the 10th International Classification of Diseases; NA, not applicable. *E. coli*, *Escherichia coli*; STEC, Shiga toxin–producing *Escherichia coli*

### Deaths

For circa 2010, we estimated that there were 60 deaths (90% CrI 53–63) due to foodborne gastroenteritis and 16 deaths (90% CrI 10–21) due to nongastrointestinal foodborne illnesses (Table 4). Nontyphoidal *Salmonella* spp. and *L. monocytogenes* were the most commonly identified causes of all illnesses that resulted in death; each year, these pathogens were each responsible for an estimated 15 foodborne illness–associated deaths. Gastroenteritis of unknown etiology as an underlying or contributing cause of death resulted in 39 deaths each year.

## Discussion

Foodborne illness is extremely common in Australia: on average, each person in Australia experiences an episode of foodborne gastroenteritis approximately every 5 years. Although foodborne gastroenteritis is often not serious, the cost to society is considerable through direct medical costs and days of lost work. Approximately 1 in 5 persons with gastroenteritis seeks medical attention. Thus, up to 1 million medical visits a year could be for foodborne illnesses (*23*).

We examined changes in foodborne illness in Australia over time, a key reason for repeating studies to estimate incidence. Our findings showed a slight decline in the rate of foodborne gastroenteritis between the circa 2000 and circa 2010 study periods, but our findings also showed increases in the rates of illness caused by some specific pathogens. Changed estimates were driven by differences in estimates of total gastroenteritis and by pathogen-specific surveillance trends. In Australia from 2006 onward, the number of raw egg–associated salmonellosis outbreaks has markedly increased (*24*), and since 2000, the numbers of notified laboratory-confirmed cases of campylobacteriosis and salmonellosis have increased (*25*). Estimates of rotavirus cases for circa 2010 were lower than those for circa 2000, reflecting the success of the vaccination program (*18*). Also, the estimated number of foodborne illness cases caused by hepatitis A virus declined from 150 cases/year circa 2000 to 40 cases/year circa 2010, reflecting improved disease control through vaccination (*24*). Although these interventions were not targeted at foodborne disease, our findings highlight the benefits of vaccination programs in reducing circulation of enteric pathogens and transmission through food.

It must be noted that where we observed changes over time, they were often not significant due to the many sources of uncertainty. When we examined the CrIs, over half of the uncertainty arose from the distribution for the foodborne multiplier estimated from expert elicitation; most of the other sources of uncertainty arose from the distributions for the underreporting and pathogen fraction multipliers. Further studies to estimate foodborne multipliers for high-incidence pathogens (in particular, norovirus and other pathogenic *E. coli*) would help reduce this uncertainty in overall estimates. Scallan et al*.* (*3*) highlighted the profound effect that changes in these proportions of foodborne transmission can have on overall estimates of disease incidence. We identified similar effects when we used updated methods to recalculate estimates for circa 2000; in particular, the estimates for foodborne gastroenteritis illnesses declined from 5.4 to 4.3 million cases. New approaches should be examined for estimating the relative importance of different modes of transmission for pathogens that are potentially foodborne.

Similar studies estimating the incidence of foodborne disease have been conducted in the United States (*2*,*3*,*17*), United Kingdom (*4*), Canada (*22*), and the Netherlands (*5*). We estimated that 25% of all gastroenteritis cases in Australia were caused by contaminated food; this percentage is similar to estimates for the United Kingdom and to the most recent estimates for the United States but lower than estimates for the Netherlands. Although the Canadian study does not report an overall proportion of foodborne transmission, analysis of the study results puts it at ≈20% (*22*). In the United States, Scallan et al. (*2*) estimated that 9.4 million (26%) of 36.4 million domestically acquired illnesses caused by known pathogens were transmitted via contaminated food, and in the United Kingdom, Adak et al. (*4*) estimated that 26% of infectious intestinal illnesses were caused by pathogens transmitted via contaminated food. The estimate for the Netherlands was higher at 39% (*5*). These overall estimates of the proportion of gastroenteritis caused by contaminated food depend on the pathogens included in the estimates, the incidence of common pathogens in the study area, and the proportion of those common pathogens that are considered to be foodborne.

The methods we used to calculate estimates in this study were refined from those used for the circa 2000 study, and in the intervening years, surveillance has improved and data availability has increased. In addition, we used national data to incorporate variations in foodborne disease patterns to provide more representative estimates. A further improvement was our use of more detailed hospitalization data. Previous hospitalization estimates for foodborne gastroenteritis were determined by using the hospital principal diagnosis data with a multiplier to adjust for additional diagnoses. In this study, we used the principal plus additional diagnoses data so that we could identify different diagnosis patterns by pathogen; for example, we found that 77% of the hospital diagnoses for salmonellosis were listed as principal diagnoses, whereas 37% of the diagnoses for norovirus infection were listed as principal diagnoses. Our new approach better captures different diagnosis patterns, especially for illnesses with multiple concomitant conditions (e.g., listeriosis) (*26*).

We also incorporated new expert elicitations into our methods to determine the circa 2010 estimates, further improving data quality (*19*). These expert elicitations were undertaken in 2009 to decide which pathogens/agents should be included in the estimates and to determine the proportion of cases caused by foodborne transmission. Compared with estimates obtained by using the Delphi process in 2005 (*21*), the estimated proportion of foodborne transmission in the circa 2010 study was generally lower, and uncertainty bounds were generally wider. In particular, our estimates showed a lower proportion of foodborne transmission for *Clostridium perfringens*, other pathogenic *E.* *coli*, norovirus, nontyphoidal *Salmonella* spp., and Shiga toxin–producing *E. coli* (STEC). This finding may reflect that environmental sources of gastrointestinal infection have been somewhat neglected and that health departments have a primary focus on foodborne diseases (*19*). Compared with previously published estimates for 2000 (*7*), our estimates for circa 2000 showed fewer illnesses attributed to food; this difference was due to our use of lower foodborne proportions for some pathogens.

When estimating the community incidence of foodborne illness, we used underreporting multipliers to adjust for the proportion of infected persons who did not seek treatment or submit specimens for testing. We used previously published estimates (*16*) of pathogen-specific multipliers for nontyphoidal *Salmonella* spp., *Campylobacter* spp., and STEC. The underreporting multiplier used for nontyphoidal *Salmonella* spp. (7, 95% CrI 4–14) was extrapolated to all other moderate illnesses, except *Campylobacter* spp. and STEC. These new underreporting multipliers were smaller than those used in previously published estimates for Australia (15, 95% CrI 5–25) (*7*).

The underreporting multiplier for serious illnesses and the underdiagnosis multiplier for hospitalizations and deaths remained at 2 (CrI 1–3), consistent with usage in other studies (*2*,*17*,*27*). The use of this multiplier for hospitalizations and deaths was validated by comparing data from the OzFoodNet Outbreak Register with hospital and death data, which suggested that a multiplier of at least 2 was necessary to account for underdiagnosis. Data on pathogen-specific underdiagnosis are limited, and further studies are required to thoroughly validate this multiplier and assess whether there are pathogen-specific differences in the underdiagnosis of severe illness.

The incidence of cases, hospitalizations, and deaths associated with foodborne pathogens in Australia does not show the complete burden from these pathogens because infection with some of them (i.e., *Campylobacter* spp., nontyphoidal *Salmonella* spp., and STEC) may lead to sequelae. The estimates in this study, together with our estimates of sequelae (*8*), highlight the considerable effect of foodborne *Campylobacter* spp. infection in Australia (*28*).

In a complex study of this type, there are several gaps and limitations in the data. While NNDSS and the OzFoodNet Outbreak Register are nationally representative, jurisdictions may have reported or coded their data differently. In addition, there were no available Australian data on toxoplasmosis, so we relied on data from the United States (*29*). We used data from the WQS (*10*,*11*) for pathogens that were not nationally notifiable or had limited outbreak data. The WQS study was the best of its kind in Australia; however, the data are now >15 years old, and the study population was based on families in Melbourne with children. We adjusted WQS data for changes over time and weighted the data for the age structure of the general population (Technical Appendix 2). In addition, cohort study participants may be reluctant to provide fecal samples; in the WQS, only one third of persons with gastroenteritis submitted a fecal sample (*11*). Furthermore, the WQS did not test for all known foodborne pathogens, and a pathogen was identified for only 17% of the fecal specimens that were examined (*10*).

The estimated incidence of foodborne disease in Australia circa 2010 was considerable: 4.1 million cases (90% CrI 2.3–6.4) of foodborne gastroenteritis and 5,140 cases (90% CrI 3,530–7,980) of nongastrointestinal foodborne illness occurred annually. Most foodborne illness occurs as gastroenteritis, but the effect of nongastrointestinal illnesses and sequelae are substantial because they can result in hospitalization and, occasionally, death. We identified that over time, the incidence of all foodborne gastroenteritis declined, but the incidences of salmonellosis and campylobacteriosis increased, although changes were not significant due to amount of uncertainty inherent in our estimates. These findings should assist policy makers to advocate for improved regulation and control of foodborne disease for specific pathogens.

Technical Appendix 1 Data sources for estimates of foodborne illness in Australia, circa 2000 and circa 2010.

Technical Appendix 2 Methods for calculating the incidence of foodborne illnesses in the community circa 2010 and for comparing those estimates with circa 2000 estimates, Australia.

Technical Appendix 3 Methods used to estimate hospitalizations and deaths due to foodborne illnesses, Australia, circa 2000 and circa 2010.

Technical Appendix 4 Pathogen and illness sheets for foodborne illnesses, Australia, circa 2000 and circa 2010.

Technical Appendix 5 Median numbers of gastroenteritis and nongastrointestinal illness cases caused by all domestically acquired pathogens and by domestically acquired foodborne pathogens, Australia, circa 2010.
