# Supplementary material for: Foodborne Illness, Australia, Circa 2000 and Circa 2010
Source: Emerg Infect Dis. 2014 Nov;20(11):1857–64. doi: 10.3201/eid2011.131315 (PMC4214288; doi:10.3201/eid2011.131315)
Supplement: Technical Appendix 1 — Data sources for estimates of foodborne illness in Australia, circa 2000 and circa 2010. [file 13-1315-Techapp-s1.pdf]

Article DOI: http://dx.doi.org/10.3201/eid2011.131315

Foodborne Illness, Australia, Circa 2000 and Circa 2010

Technical Appendix 1

Data Sources

Estimates of illness based on surveillance data used notifiable surveillance data at the national or State level or other surveillance through the OzFoodNet Outbreak Register. Estimates of incidence were also calculated based on the 2008 Australian National Gastroenteritis Survey (NGSII) together with a fractional pathogen approach derived from cohort studies, such as the Water Quality Study (*1*–*3*). The data source and estimation approach used for each pathogen is explained in the Table.

Technical Appendix 1 Table. Data sources and estimation approach used for each pathogen or syndrome*

Pathogen or illness	Data Source	Estimation Approach
Campylobacter spp.	NNDSS	Notifiable Surveillance
Salmonella spp., nontyphoidal†	NNDSS	Notifiable Surveillance
Salmonella enterica serotype Typhi	NNDSS	Notifiable Surveillance
Shigella spp.	NNDSS	Notifiable Surveillance
Cryptosporidium spp.	NNDSS	Notifiable Surveillance
Hepatitis A	NNDSS	Notifiable Surveillance
Listeria monocytogenes	NNDSS	Notifiable Surveillance
Giardia lamblia	State Surveillance	Notifiable Surveillance
STEC	State Surveillance	Notifiable Surveillance
Vibrio parahaemolyticus	State Surveillance	Notifiable Surveillance
Yersinia enterocolitica	State Surveillance	Notifiable Surveillance
Other pathogenic *Escherichia coli*	NGSII (*1*) and WQS (*2*,*3*)	Pathogen Fraction
Adenovirus	NGSII (*1*) and WQS (*2*,*3*)	Pathogen Fraction
Astrovirus	NGSII (*1*) and WQS (*2*,*3*)	Pathogen Fraction
Norovirus	NGSII (*1*) and WQS (*2*,*3*)	Pathogen Fraction
Rotavirus	NGSII (*1*) and WQS (*2*,*3*)	Pathogen Fraction
Sapovirus	NGSII (*1*) and WQS (*2*,*3*)	Pathogen Fraction
Bacillus cereus	OzFoodNet Outbreak Register	Other Surveillance
Clostridium perfringens	OzFoodNet Outbreak Register	Other Surveillance
Staphylococcus aureus	OzFoodNet Outbreak Register	Other Surveillance
Ciguatera	OzFoodNet Outbreak Register	Other Surveillance
Scombrotoxicosis	OzFoodNet Outbreak Register	Other Surveillance
Toxoplasma gondii	U.S. Seroprevalence Study (*4*)	Special Calculations

*NGSII, National Gastroenteritis Survey II; NNDSS, National Notifiable Disease Surveillance System; STEC, Shiga toxin–producing *Escherichia coli;* WQS, Water Quality Study.
†Refers to nontyphoidal *Salmonella enterica* serotypes.

Notifiable Surveillance: National Notifiable Disease Surveillance Scheme and State Notifications

The Australian National Notifiable Disease Surveillance System (NNDSS) provides national data for pathogens that are notifiable in Australia, such as *Salmonella* spp., *Shigella* spp. and *Cryptosporidium* spp. Some pathogens are notifiable in some States, but not in others; for

example, *Campylobacter* spp. is not notifiable in New South Wales, but is notifiable in all other States. In these cases, we use notification data for the available States and included a population adjustment multiplier to estimate national notification rates (see online Technical Appendix 2, http://wwwnc.cdc.gov/EID/article/20/11/13-1315-Techapp2.pdf). In each case, we have used the total number of confirmed notifications for all available years over the period 2006–2010.

Additionally, we requested further data through the Communicable Disease Network of Australia (CDNA) to determine the proportion of cases that were domestically acquired in Australia. Details of the use of these data are described in online Technical Appendix 2 under the section title Domestically Acquired Multiplier.

Other Surveillance: OzFoodNet Outbreak Register

The OzFoodNet Outbreak Register includes all outbreaks identified over the period 2006–2008, providing data on the number of persons ill in each outbreak, the pathogen identified, and the total number of persons with laboratory confirmed illness in each outbreak.

National Gastroenteritis Survey II 2008

The NGSII was a nationally representative telephone survey conducted by the Department of Health and Ageing, the New South Wales Food Authority and the National Centre for Epidemiology and Population health in 2008–2009 to improve estimates of burden of gastroenteritis in Australia. It provides age-specific rates of gastroenteritis in the community.

Research Studies

We used Australian and international cohort studies to assess the proportion of gastroenteritis that is due to specific pathogens. A key study is the 1997 Water Quality Survey, which was a double-blinded, randomized, controlled trial of families conducted in Melbourne, Australia between September 1997 and February 1999 (*2*,*3*). Six hundred families were allocated to receive either real or sham water treatment units installed in their houses and study participants reported any gastroenteritis symptoms weekly. The study provides testing data on 795 fecal specimens identifying pathogens causing gastroenteritis, and we used this data to calculate a pathogen fraction multiplier for included pathogens (online Technical Appendix 2). As there was no significant difference in incidence of gastroenteritis in control and experimental families, the study found that waterborne pathogens do not play a major role in gastroenteritis in Melbourne (*2*).

References

1. Kirk M, McKercher C, Hall G. Gastroenteritis in Australia: report of the National Gastroenteritis Survey II 2008. Canberra (Australia): OzFoodNet and the National Centre for Epidemiology and Population Health; 2011 Feb.

2. Hellard ME, Sinclair MI, Forbes A, Fairley CK. A randomized, blinded, controlled trial investigating the gastrointestinal health effects of drinking water quality. Environ Health Perspect. 2001;109:773–8. PubMed http://dx.doi.org/10.1289/ehp.01109773

3. Sinclair MI, Hellard ME, Wolfe R, Mitakakis TZ, Leder K, Fairley CK. Pathogens causing community gastroenteritis in Australia. J Gastroenterol Hepatol. 2005;20:1685–90. PubMed http://dx.doi.org/10.1111/j.1440-1746.2005.04047.x

4. Jones JL, Kruszon-Moran D, Sanders-Lewis K, Wilson M. *Toxoplasma gondii* infection in the United States, 1999–2004, decline from the prior decade. Am J Trop Med Hyg. 2007;77:405–10. PubMed
