# Supplementary material for: Foodborne Illness, Australia, Circa 2000 and Circa 2010
Source: Emerg Infect Dis. 2014 Nov;20(11):1857–64. doi: 10.3201/eid2011.131315 (PMC4214288; doi:10.3201/eid2011.131315)
Supplement: Technical Appendix 2 — Methods for calculating the incidence of foodborne illnesses in the community circa 2010 and for comparing those estimates with circa 2000 estimates, Australia. [file 13-1315-Techapp-s2.pdf]

Article DOI: http://dx.doi.org/10.3201/eid2011.131315

Foodborne Illness, Australia, Circa 2000 and Circa 2010

Technical Appendix 2

Calculating Community Incidence

Approaches and Distributions

We adopted three main approaches to calculating the incidence of illness in the community. These three approaches are based on the source of the data as

1. Notifiable surveillance approach using data from the National Notifiable Diseases Surveillance System (NNDSS) or State notifications;
2. Pathogen fraction approach using data from the 2008 National Gastroenteritis Survey II (NGSII) together with cohort studies, such as the Water Quality Study;
3. Other surveillance approach using data from the OzFoodNet Outbreak Register, or from hospitalizations

We considered these approaches to form a hierarchy, with the notifiable surveillance approach used by preference, and outbreak data used only when other sources were not available. For each approach, the final estimate was produced from a statistical model that incorporates uncertainty in case numbers and in multipliers using probability distributions. That is, at each stage of calculation, the estimate was represented by a probability distribution, and our final estimates and credible intervals were computed from these distributions. Where data for multiple approaches were available, we computed both and used the lower-hierarchy estimates as an informal cross-check.

Figures 1, 2, and 3 provide flowcharts explaining this approach. In each flowchart, the left-hand column provides a description of each input or output distribution, the central column provides a pictorial representation of the distribution, and the right-hand column describes the

type and source of data underlying each input distribution. In each case, input data arises from specific data sources (discussed in online Technical Appendix 1, http://wwwnc.cdc.gov/EID/article/20/11/13-1315-Techapp1.pdf), or from multipliers discussed below. We used three main input distribution types: empirical, PERT, and lognormal.

Empirical Distribution

Source distributions on the number of cases were typically represented by an empirical or discrete distribution driven by the data. For example, if the number of cases notified to NNDSS for the years 2006–2010 were 15416, 16980, 15539, 16075, and 16967, we would represent this as a discrete distribution with 20% of the probability mass at 15416, 20% of the probability mass at 16980, and so on. This use of empirical distributions for such data was used previously by Scallan et al. (*1*), and allowed us to avoid any assumptions about the expected shape of the distribution.

PERT Distribution

The PERT distribution is widely used for expert elicitation and risk assessment studies. It is based on the β distribution, and within the computer software @Risk, can be specified either using a minimum, maximum and modal value, or by three percentile points, such as a median value and 95% credible intervals. We used this distribution widely in our analysis, as it allows for asymmetric distributions, and can be easily produced from many data sources including expert elicitation

Lognormal Distribution

When re-calculating our underreporting multipliers we discovered that the PERT distribution did not adequately capture the shape of these multipliers. We adopted a lognormal distribution instead, as the distribution providing the best fit as measured by @Risk, and demonstrating an improved fit on the normal distribution used previously (*2*).

Multipliers

Figures 1, 2, and 3 give flowcharts for calculating foodborne disease illness using key multipliers either to scale up (surveillance approaches) from detected cases to the full community burden, or to scale down (pathogen fraction approach) from all gastroenteritis to the proportion that is due to specific pathogens.

Population Adjustment Multiplier

This multiplier was used where notifiable surveillance data are not available for all States in Australia, and was necessary to scale up the number of infections according to the proportion of the population covered by the surveillance data. For example, *Campylobacter* spp. is notifiable in all States except New South Wales. In this example we adjusted our total number of cases for the remaining States by a population adjustment multiplier of 1.5 to approximate the total number of cases we would expect to see if all States undertook notifiable surveillance.

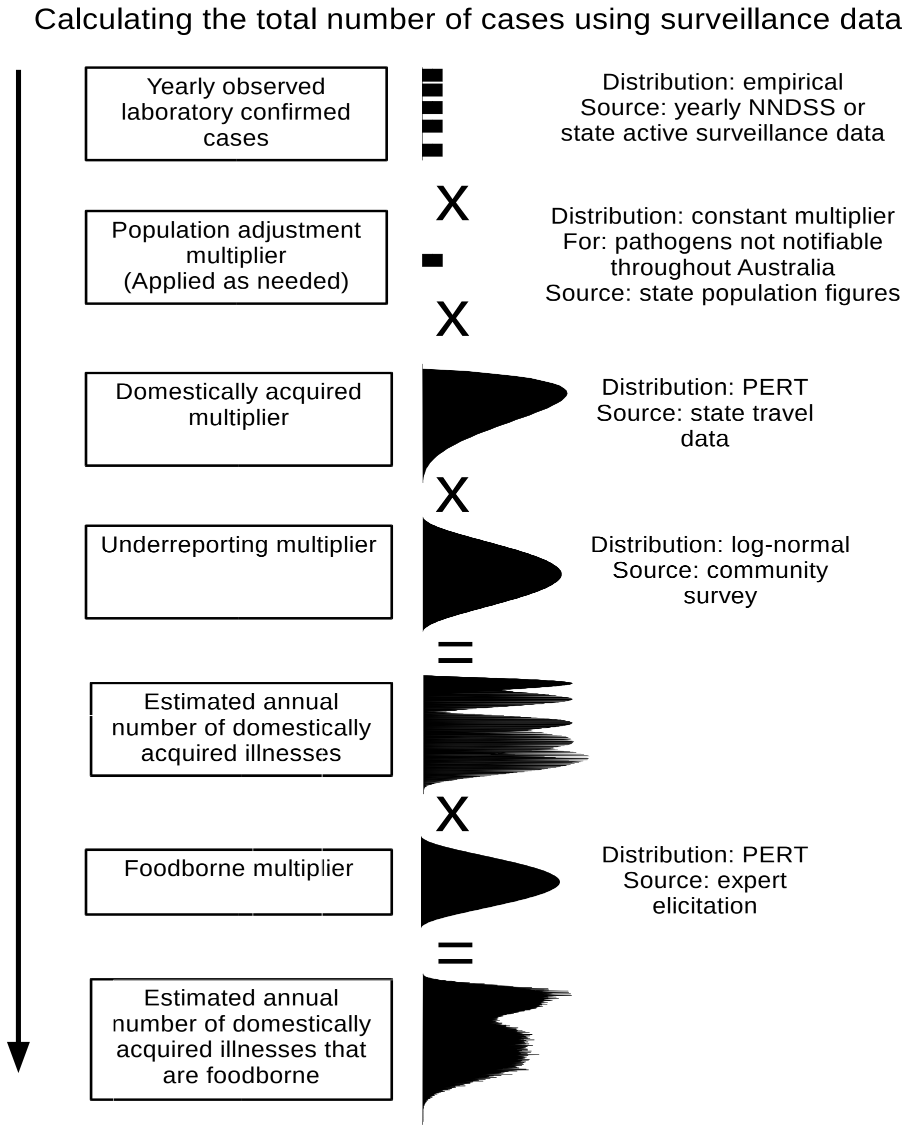

Technical Appendix 2 Figure 1. Flowchart for the notifiable surveillance approach used to calculate the estimated annual number of domestically acquired illnesses that are foodborne.

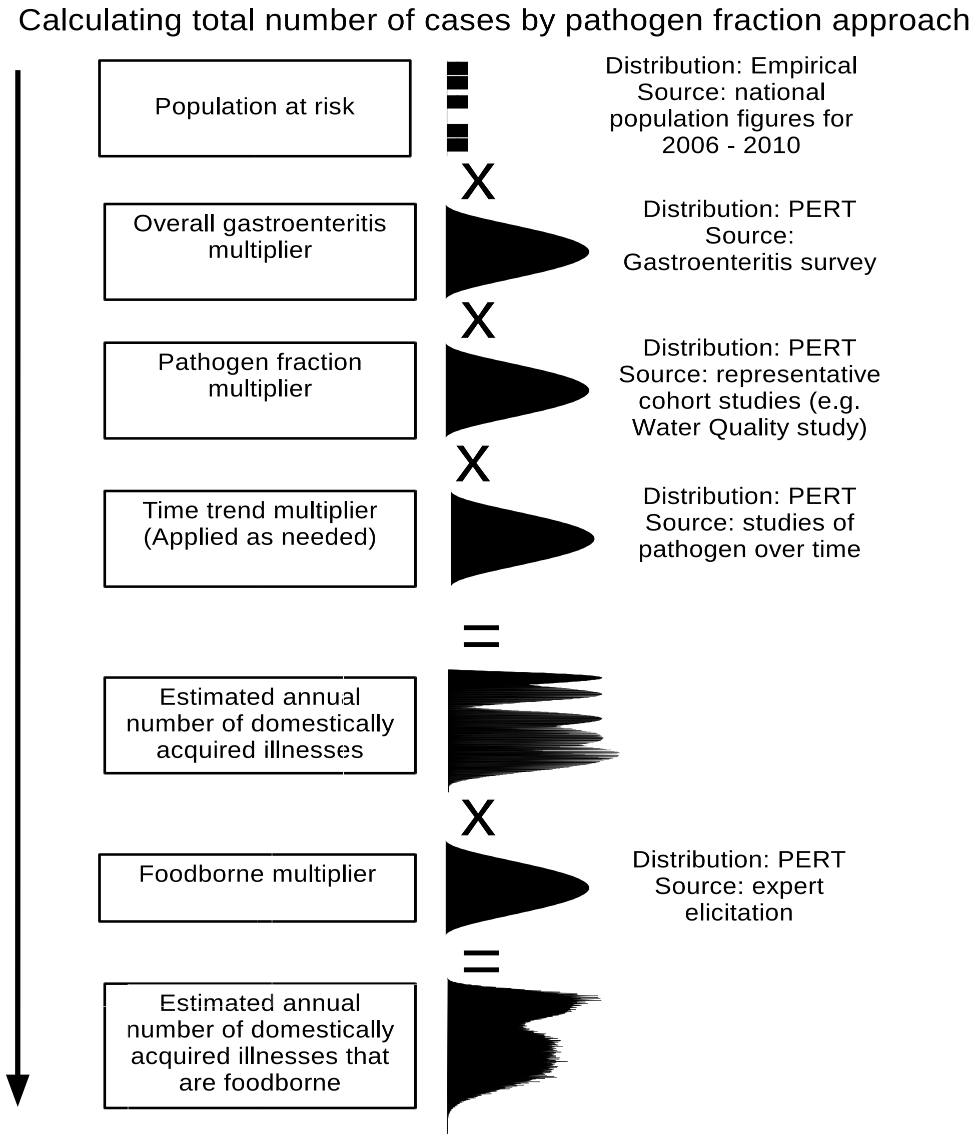

Technical Appendix 2 Figure 2. Flowchart for the pathogen fraction approach used to calculate the estimated annual number of domestically acquired illnesses that are foodborne.

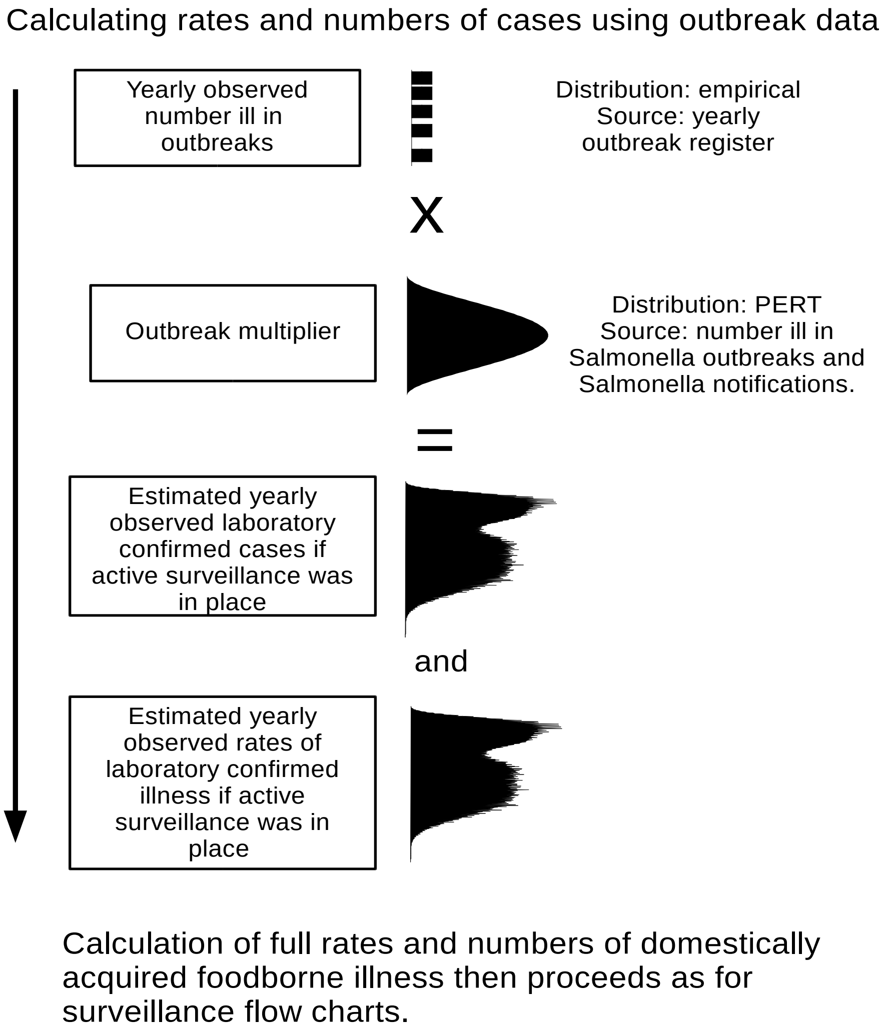

Technical Appendix 2 Figure 3. Flowchart for the other surveillance approach used to calculate the estimated annual number of domestically acquired illnesses that are foodborne.

Domestically Acquired Multiplier

For some pathogens, a proportion of cases acquired their infections overseas. As data from the Water Quality Study used for the pathogen fraction calculations was centered on families, we assumed all these incident cases were domestically acquired. For *Campylobacter* spp., *Cryptosporidium* spp., hepatitis A, *Listeria monocytogenes*, nontyphoidal *Salmonella enterica* serotypes (hereafter referred to as nontyphoidal *Salmonella* spp.), *Salmonella enterica* serotype Typhi, *Shigella* spp., and Shiga toxin-producing *Escherichia coli* (STEC), the domestically acquired multiplier was calculated from NNDSS data on the proportion of cases that acquired their infection within Australia. This data contained several missing entries,

varying by pathogen, State and year, with the most complete data for Victoria and Western Australia. We considered four methods for adjusting for this missing data:

1. Extrapolate travel patterns from Western Australia to the Northern Territory and travel patterns from Victoria to all other States;
2. Extrapolate travel patterns from Western Australia to both the Northern Territory and Queensland, and travel patterns from Victoria to all other States;
3. Discard all missing data and calculate the proportion of cases acquired in Australia for the existing data only;
4. Assume all unidentified cases are domestically acquired

We adopted method 1 as the primary approach, and used the other methods as a comparison and to identify an uncertainty range for the multiplier. Specifically, the median estimate was made using all 5 years of data combined, while the minimum and maximum value reflects the largest and smallest proportion estimated by all four methods over each year of 2006–2010. Table 1 presents the resulting parameters for the PERT distribution, including median value, minimum and maximum, together with the estimations used by Hall et al. (*3*) for Australian estimates circa 2000. For *Cryptosporidium* spp., nontyphoidal *Salmonella* spp., and *Shigella* spp., estimates on the full data over 2006–2010 using methods 1 and 3 were reassuringly similar, while the expanded ranges reflect the yearly variability and sensitivity to missing data. Larger differences are seen for hepatitis A, *S. enterica* serotype Typhi, and STEC. There were very few missing data for hepatitis A and *S. enterica* serotype Typhi which raises our confidence in these estimates. Only 0 to 2 overseas cases of STEC were recorded per year, and this is reflected in the higher estimate of domestically acquired infection for this pathogen. This multiplier was also used for calculations of hospitalizations and deaths for other pathogenic *Escherichia coli*.

Estimates for the domestically acquired multiplier for *Giardia lamblia* were made using Victorian data over 2006–2009 (*4*–*7*), using the total proportion to derive the median and the variability over years to give a range. Domestically acquired multipliers for *Vibrio parahaemolyticus* and *Yersinia enterocolitica* were calculated from Western Australian data in a similar manner using OzFoodNet Annual Reports from 2006–2010. Given the higher rate of

overseas acquired infections in WA as compared with other jurisdictions, we reduced the proportion overseas for other States using a multiplier of 0.72 based on data for nontyphoidal *Salmonella* spp. Even with this adjustment, the multiplier for *V. parahemolyticus* is much lower than that used in the U.S. suggesting a greater proportion of overseas-acquired cases in Australia (*1*); more information on the behavior of this pathogen in States outside Western Australia would be valuable to confirm our results.

Finally, we assumed that all cases of adenovirus, *Bacillus cereus*, ciguatera, *Clostridium perfringens, L. monocytogenes*, norovirus, rotavirus, scombrotoxicosis, *Staphylococcus aureus*, and *Toxoplasma gondii* were acquired in Australia. Domestically acquired multipliers were not needed for the remaining pathogens (astrovirus and sapovirus) for which incidence was calculated using the pathogen fraction approach, and that do not have specific codes to calculate hospitalizations and deaths.

Technical Appendix 2 Table 1. Estimated proportion of domestically acquired foodborne infections circa 2010 compared with previously published estimates for circa 2000, Australia*

	Estimated % (range) of domestically acquired foodborne illnesses	
Pathogen or Illness	Circa 2010	Circa 2000
Adenovirus	100 (100–100)	
Bacillus cereus	100 (100–100)	
Campylobacter spp.	97 (91–99)	96
Ciguatera	100 (100–100)	
Clostridium perfringens	100 (100–100)	
Cryptosporidium spp.	97 (92–99)	
Giardia lamblia	85 (84–89)	
Hepatitis A	58 (42–77)	
Listeria monocytogenes	100 (100–100)	
Norovirus	100 (100–100)	
Other pathogenic *Escherichia coli*	99 (93–100)	
Rotavirus	100 (100–100)	
Salmonella spp., nontyphoidal†	85 (70–95)	92
Salmonella enterica serotype Typhi	11 (2–25)	
Scombrotoxicosis	100 (100–100)	
Shigella spp.	70 (45–84)	60
Staphylococcus aureus	100 (100–100)	
STEC	99 (93–100)	79
Toxoplasma gondii	100 (100–100)	
Vibrio parahaemolyticus	18 (0–33)	
Yersinia enterocolitica	90 (80–100)	98

*Data circa 2000 was obtained for select pathogens and illnesses from 2 states (Victoria and South Australia) (*8*) Range was not provided. STEC, Shiga toxin–-producing *Escherichia coli*.
†Refers to nontyphoidal *Salmonella enterica* serotypes.

Underreporting Multiplier

Only a fraction of community cases visit a health professional, have a sample taken and have their illness recorded in surveillance data. Using data from Hall et al. (*2*), we estimated underreporting multipliers based on lognormal distributions of 7 (95% Credible Interval 4–14) for nontyphoidal *Salmonella* spp., 10 (95% CrI 6.5–18.5) for *Campylobacter* spp., and 8 (95% CrI 3–18.5) for STEC. Where underreporting multipliers were needed for other pathogens, we

applied the nontyphoidal *Salmonella* spp. multiplier except in the case of pathogens leading to very severe illness (hepatitis A, *L. monocytogenes,* and *S. enterica* serotype Typhi) where the underreporting multiplier was assumed to be 2 (95% CrI 1–3). Details of the choice of multiplier for each pathogen are provided in online Technical Appendix 4 (http://wwwnc.cdc.gov/EID/article/20/11/13-1315-Techapp4.pdf).

Foodborne Multiplier

For most pathogens, we estimated the proportion of illness that is foodborne using data from Delphi based expert elicitations. For nine pathogens, we used a 2009 elicitation, and for another eight, we used a similar 2005 elicitation (*9*). The 2009 elicitation was informed by systematic reviews for each pathogen that included scientific literature, reports and surveillance data. Eleven experts estimated the proportion of illness transmitted via food through three rounds: the first round taking place after training questions, the second round after they had been provided with systematic reviews for all pathogens, and the final round after a 1-day workshop in which experts discussed each pathogen. At each step, experts were asked to estimate the proportion of transmission that is due to food, environment, water, animal or person-to-person transmission, making sure that these proportions summed to 1. The experts were then asked to give 90% certainty bounds for their foodborne proportion. Foodborne proportion estimates and intervals from the final stage of the elicitation were combined using PERT distributions. We extrapolated sapovirus from elicited norovirus estimates, and used best judgment assumptions for three additional viruses and the two marine biotoxins. See Table 2 for a listing of pathogens, multipliers and the data source for each. A comparison of these estimates with those used in prior studies is provided elsewhere (*9*).

Expert elicitation data from 2009 includes a best estimate and 90% interval for *Campylobacter* spp., *C. perfringens,* STEC, other pathogenic *E. coli*, nontyphoidal *Salmonella* spp., *Shigella* spp., norovirus, hepatitis A, and *L. monocytogenes*. We fitted a PERT distribution to each expert’s assessment, fitting the best estimate as the median and setting the 90% interval where possible. In a few cases, we could not fit a PERT distribution in this way, and either had to adjust the best estimate to be the mode of the distribution (if the median point was two close to an upper or lower bound), or adjust an interval bound to be a min or max if the PERT distribution led to values outside the interval 0 to 1. A combined empirical distribution was

calculated by computing the point-wise mean value of the individual uncertainty distribution for each expert. The median, 5% and 95% percentiles of this empirical distribution were then used to describe a final PERT distribution that was input into the relevant @Risk spreadsheet.

The 2005 questionnaire provided a best estimate from participants. To include uncertainty in this estimate, we generated a 90% credible interval about each estimate, assuming an upper bound 10 percentage points higher and a lower bound 10 percentage points lower. For example, an estimate of 30% foodborne was modeled as a PERT distribution with median as 0.3, 95% bound 0.4, and 5% bound 0.2. The exception to this was where estimates were too close to zero (or one) for this method. We then assumed symmetric estimates half the distance from zero (or one). That is, an estimate of 5% foodborne was modeled as a PERT with median as 0.05, 5% bound as 0.025 and 95% bound as 0.075. The combined distribution was calculated as for the expert elicitation data. The 2005 elicitation did not achieve consensus for some pathogens; in particular, best estimates ranged from 2%–95% for *S. enterica* serotype Typhi, 5%–100% for *V. parahemolyticus*, and 33%–90% for *Y. enterocolitica*. Given the variability arising from these expert data, we tested the sensitivity of our results to the choice of distribution by simulating the full empirical distribution of the foodborne multiplier for each of these pathogens, and compared estimates of foodborne illness with those using the PERT distribution. In general, median estimates were little changed, but credible intervals were a little wider under the empirical distribution. The largest change was for *Y. enterocolitica*, where the estimate of domestically acquired foodborne illness was 1,150 (650–1950) using a PERT distribution, and 1,100 (350–2,050) using the empirical distribution.

Outbreak Multiplier

For pathogens that are not captured by notifiable surveillance or by cohort studies, we used data from outbreaks in the other surveillance approach. Only a fraction of cases are associated with outbreaks. The outbreak multiplier adjusts for this to estimate the total number of cases that would be captured if notifiable surveillance was in place for that pathogen. Many of the pathogens for which this method was used have a short duration of illness, and thus low rates of laboratory confirmation. To adjust for this, we calculated the multiplier based on total number of ill (but not necessarily lab confirmed) cases associated with a confirmed outbreak (where laboratory confirmation of at least one case or of a food source has been occurred). We chose to

use nontyphoidal *Salmonella* as the reference pathogen for the outbreak multiplier as it has the most complete data. The outbreak multiplier was calculated as the ratio of the number of ill cases in outbreaks of nontyphoidal *Salmonella* spp. to the total number of laboratory confirmed domestically acquired cases of nontyphoidal *Salmonella* spp. in the NNDSS for the same year. For example, in 2008 there were 8, 316 laboratory confirmed cases of nontyphoidal *Salmonella* spp. in NNDSS, of which 85% (range: 70–90) were assumed to be acquired in Australia. The total number of ill cases associated with nontyphoidal *Salmonella* spp. outbreaks in 2008 was 524, giving an outbreak multiplier of around 13.5 for this year. Extending this approach to calculate multipliers for each year from 2006–2008, and for data for all years combined, we estimate an outbreak multiplier of 14, with range 5–20.

Gastroenteritis Multiplier

For pathogens captured by cohort studies such as the Water Quality Study (*10*,*11*), we attributed a proportion of all gastroenteritis cases to that pathogen using the pathogen fraction approach (see Figure 2). The first step of this approach was to determine the total incidence of gastroenteritis. To do this we used the NGSII study to estimate the total number of gastroenteritis episodes per person per year, weighted by the Australian population. This estimate served to provide a gastroenteritis multiplier, which was then multiplied by the total Australian population for the years 2006–2010 to give the estimated number of cases of gastroenteritis for each year. The gastroenteritis multiplier was modeled as an alternative PERT distribution with median 0.74 and 95% interval (0.64–0.84), based on the estimates and uncertainty intervals estimated by the NGSII study.

Technical Appendix 2 Table 2. Estimates of the foodborne multiplier with 90% credible interval using PERT distributions for each of the 23 pathogens*

Pathogen or Illness	Foodborne multiplier (90% CrI)†	Data source‡
Adenovirus	0.02 (0.01–0.03)	Assumption
Astrovirus	0.02 (0.01–0.03)	Assumption
Bacillus cereus	1.00 (0.98–1.00)	2005 EE as PERT
Campylobacter spp.	0.77 (0.62–0.89)	2009 EE as PERT
Ciguatera	1.00 (1.00–1.00)	Assumption
Clostridium perfringens	0.98 (0.86–1.0)	2009 EE as PERT
Cryptosporidium spp.	0.10 (0.01–0.27)	2005 EE as PERT
Other pathogenic *Escherichia coli*	0.23 (0.08–0.55)	2009 EE as PERT
Giardia lamblia	0.06 (0.01–0.50)	2005 EE as PERT
Hepatitis A	0.12 (0.05–0.24)	2009 EE as PERT
Listeria monocytogenes	0.98 (0.90–1.00)	2009 EE as PERT
Norovirus	0.18 (0.05–0.35)	2009 EE as PERT
Rotavirus	0.02 (0.01–0.03)	Assumption
Salmonella spp., nontyphoidal§	0.72 (0.53–0.86)	2009 EE as PERT
Salmonella enterica serotype Typhi	0.75 (0.02–0.97)	2005 EE as PERT
Sapovirus	0.18 (0.05, 0.35)	Norovirus multiplier
Scombrotoxicosis	1.00 (1.00, 1.00)	Assumption
Shigella spp.	0.12 (0.05, 0.23)	2009 EE as PERT

Pathogen or Illness	Foodborne multiplier (90% CrI)†	Data source‡
Staphylococcus aureus	1.00 (0.95, 1.00)	2005 EE as PERT
STEC	0.56 (0.32, 0.83)	2009 EE as PERT
Toxoplasma gondii	0.31 (0.04, 0.74)	2005 EE as PERT
Vibrio parahaemolyticus	0.75 (0.05, 0.96)	2005 EE as PERT
Yersinia enterocolitica	0.84 (0.28, 0.94)	2005 EE as PERT

*Program evaluation review technique (PERT) is a commonly used distribution in expert elicitation and is based on a two parameter Beta distribution. STEC, Shiga toxin–producing *Escherichia coli*.
†Credible Intervals.
‡ See Vally et al (*8*) for a comparison of these estimates with those used in prior studies. EE = Expert elicitation.
§Refers to nontyphoidal *Salmonella enterica* serotypes.

Pathogen Fraction Multiplier

The pathogen fraction multiplier attributed a proportion of the total number of gastroenteritis episodes to particular pathogens. Our primary data source for this was the Water Quality Study (*10,11*). While we also used data from the UK IID2 study (*12*) as a comparator, we found the Water Quality study gave the most reliable picture of the burden of illness due to different pathogens in Australia. The data from the study were age-adjusted (using age ranges 0–4, 5–14, 15+) to the Australian population (circa 2010) to take account of the higher numbers of children in the Water Quality study. For example, the raw data for adenovirus in the Water Quality study was 9 positive samples from a total of 713 samples taken from participants with a highly credible episode of gastroenteritis. However, 8 of those positives were from participants aged 0–4 years old, an age group over sampled in the study. Using data on the incidence of gastroenteritis by age from the NSGII study, and the Australian population as a reference, we calculated age-adjusted estimates for each pathogen based on the Water Quality Study data. For example, for adenovirus, we derived an estimate of 4 samples positive for adenovirus from 713 gastroenteritis episodes. This gave us a pathogen fraction multiplier of 0.0056 (95% CI: 0.0015–0.0143), which was then modeled in @Risk using an alternative PERT distribution. Note that the pathogen sheets provided in online Technical Appendix 4 provide the age adjusted estimates for each pathogen, so will differ slightly from studies reporting findings of the Water Quality Study.

Finally, we could not find any Australian cohort study that gave estimates of prevalence of astrovirus or sapovirus for all age groups. Instead, we used pathogen fraction multiplier from the Water Quality Study for adenovirus and norovirus, together with cohort data from children (*13*) to calculate multipliers relating astrovirus to adenovirus, and sapovirus to norovirus (*14*). Although the use of children only in this approach is not ideal, it allowed us to use Australian data. We also considered an alternative approach using data from the UK infectious intestinal disease study 2 (IID2) (*12*), but found this led to unexpectedly high estimates for astrovirus and sapovirus that were not consistent with estimates for other viral pathogens estimated using data

from the Water Quality Study (*10*,*11*). These differences perhaps arise from differences in the gastroenteritis case definitions in the UK IID2 study (*12*) and our Australian NGSII study.

Time Trend Multiplier

The Water Quality Study (*10*,*11*) was undertaken before the addition of a rotavirus vaccine to the Australian vaccination schedule in 2007. In calculating rotavirus incidence circa 2010, we included a time-trend multiplier to adjust for the reduction in rotavirus in 2010 compared with pre-vaccination levels. In calculating this multiplier, we used data from a study of rotavirus hospitalizations by age before and after the introduction of the vaccination program (*15*). By comparing age-specific hospitalization rates in 2010 with that before vaccination, we were able to estimate a time-trend multiplier of 0.34 (95% Confidence Interval 0.32–0.36) to adjust for the decline in rotavirus following vaccination.

Toxoplasmosis – Special Calculations

The calculations for toxoplasmosis differed from all other methods, as we used U.S. seroprevalence studies to estimate yearly incident cases assuming a constant force of infection with age (*16*). While there is an Australian study of toxoplasmosis (*17*), we felt the sample size was too small to rely on for this estimate. In adopting this U.S. study rather than European studies (see Pappas et al. (*18*) for a systematic review), we ensure comparability with our prior work, and take a conservative approach to estimating Australian incidence of toxoplasmosis. We then adjusted this incidence estimate by a “proportion symptomatic” multiplier of 15% (90% CrI 11–21) in line with the approach used by Hall et al circa 2000 (*3*) and that of Scallan et al. (*1*).

Comparison with estimates from 2000

Several multipliers used in these calculations have changed since our study circa 2000 (*3*). These changes do not reflect altered behavior of pathogens, but rather new knowledge and better estimates of the multipliers involved. Owing to these changed multipliers, a direct comparison of this study with that circa 2000 is misleading. To provide a more appropriate comparison, we have recalculated all estimates for 2000 using new multipliers. Our aim here is to remove components of the time comparison that we know to be misleading. As for 2010, all estimates for 2000 include all uncertainty due to (new) multipliers.

Unknown Pathogens

We used the NGSII survey of gastroenteritis conducted in 2008–2009 to estimate the total envelope of domestically acquired gastrointestinal illness, and so calculated the incidence of unknown pathogens by subtracting the incidence of known pathogens causing domestically acquired gastrointestinal pathogens from that of the survey. Credible intervals were estimated using @Risk, assuming all cases in the NGSII were domestically acquired. We calculated the foodborne multiplier for all known pathogens of 25% (90% CrI: 15–39) as a weighted average of the foodborne multiplier for each pathogen, weighted by the number of domestically acquired cases of each pathogen. Although this value is remarkably similar to that estimated by Scallan et al (*1*,*19*), it is worth noting that it is based entirely on Australian expert elicitation data, together with incidence calculations using Australian data, and so is entirely independent of that study. Examination of the two studies will identify differences in many components of the calculations. The foodborne multiplier was applied to unknown pathogens to estimate the total number of domestically acquired foodborne illness due to unknown pathogens, again using @Risk for credible intervals.

References

1. Scallan E, Hoekstra RM, Angulo FJ, Tauxe RV, Widdowson MA, Roy SL, et al. Foodborne illness acquired in the United States—major pathogens. Emerg Infect Dis. 2011;17:7–15. PubMed http://dx.doi.org/10.3201/eid1701.P11101

2. Hall G, Yohannes K, Raupach J, Becker N, Kirk M. Estimating community incidence of salmonella, campylobacter and Shiga toxin-producing *Escherichia coli* infections, Australia. Emerg Infect Dis. 2008;14:1601–9. PubMed http://dx.doi.org/10.3201/eid1410.071042

3. Hall G, Kirk M, Becker N, Gregory J, Unicomb L, Millard G, et al. Estimating foodborne gastroenteritis, Australia. Emerg Infect Dis. 2005;11:1257–64. PubMed http://dx.doi.org/10.3201/eid1108.041367

4. Brown L, Fielding J, Gregory J, Yohannes K, Higgins N, Klug G, et al. Surveillance of notifiable infectious diseases in Victoria, 2006 [cited 2012 Dec 18]. http://docs.health.vic.gov.au/docs/doc/Surveillance-of-notifiable-infectious-diseases-in-Victoria-2006

5. Brown L, El-Hayek C, Fielding J, Gregory J, Higgins N, Klug G, et al. Surveillance of notifiable infectious diseases in Victoria, 2007 [cited 2012 Dec 18]. http://docs.health.vic.gov.au/docs/doc/Surveillance-of-notifiable-infectious-diseases-in-Victoria-2007

6. Brown L, El-Hayek C, Franklin L, Gregory J, Higgins N, Klug G, et al. Surveillance of notifiable infectious diseases in Victoria [cited 2012 Dec 18]. http://docs.health.vic.gov.au/docs/doc/Surveillance-of-notifiable-infectious-diseases-in-Victoria-2008

7. Brown L, El-Hayek C, Franklin L, Gregory J, Higgins N, Klug G, et al. Surveillance of notifiable infectious diseases in Victoria, 2009 [cited 2012 Dec 18]. http://docs.health.vic.gov.au/docs/doc/Surveillance-of-notifiable-infectious-diseases-in-Victoria-2009

8. Hall G, Kirk M, Ashbolt R, Gregory J, Millard G, Raupach J, et al. How much gastroenteritis in Australia is due to food? Estimating the incidence of foodborne gastroenteritis in Australia. Canberra (Australia): National Centre for Epidemiology and Population Health; Sept 2004. NCEPH Working Paper, no. 51 [cited 2014 Aug 18]. https://digitalcollections.anu.edu.au/bitstream/1885/43182/2/WP51.pdf

9. Vally H, Glass K, Ford L, Hall GH, Kirk MD, Shadbolt C, et al. The proportion of illness transmitted by food for nine enteric pathogens in Australia: an expert elicitation. Foodborne Pathog Dis. Epub 2014 Jul 29.

10. Hellard ME, Sinclair MI, Forbes A, Fairley CK. A randomized, blinded, controlled trial investigating the gastrointestinal health effects of drinking water quality. Environ Health Perspect. 2001;109:773–8. PubMed http://dx.doi.org/10.1289/ehp.01109773

11. Sinclair MI, Hellard ME, Wolfe R, Mitakakis TZ, Leder K, Fairley CK. Pathogens causing community gastroenteritis in Australia. J Gastroenterol Hepatol. 2005;20:1685–90. PubMed http://dx.doi.org/10.1111/j.1440-1746.2005.04047.x

12. Tam CC, Rodrigues L, Viviani L, Dodds J, Evans M, Hunter P, et al. Longitudinal study of infectious intestinal disease in the UK (IID2 study): incidence in the community and presenting to general practice. Gut. 2012;61:69–77. PubMed http://dx.doi.org/10.1136/gut.2011.238386

13. McIver CJ, Hansman G, White P, Doultree JC, Catton M, Rawlinson WD. Diagnosis of enteric pathogens in children with gastroenteritis. Pathology. 2001;33:353–8. PubMed

14. Kirkwood CD, Clark R, Bogdanovic-Sakran N, Bishop RF. A 5-year study of the prevalence and genetic diversity of human caliciviruses associated with sporadic cases of acute gastroenteritis in young children admitted to hospital in Melbourne, Australia (1998–2002). J Med Virol. 2005;77:96–101. PubMed http://dx.doi.org/10.1002/jmv.20419

15. Dey A, Wang H, Menzies R, Macartney K. Changes in hospitalisations for acute gastroenteritis in Australia after the national rotavirus vaccination program. Med J Aust. 2012;197:453–7. PubMed http://dx.doi.org/10.5694/mja12.10062

16. Jones JL, Kruszon-Moran D, Sanders-Lewis K, Wilson M. *Toxoplasma gondii* infection in the United States, 1999–2004, decline from the prior decade. Am J Trop Med Hyg. 2007;77:405–10. PubMed

17. Karunajeewa H, Siebert D, Hammond R, Garland S, Kelly H. Seroprevalence of varicella zoster virus parvovirus B19 and *Toxoplasma gondii* in a Melbourne obstetric population: implications for management. Aust N Z J Obstet Gynaecol. 2001;41:23–8. PubMed http://dx.doi.org/10.1111/j.1479-828X.2001.tb01289.x

18. Pappas G, Roussos N, Falagas ME. Toxoplasmosis snapshots: Global status of *Toxoplasma gondii* seroprevalence and implications for pregnancy and congenital toxoplasmosis. Int J Parasitol. 2009;39:1385–94. PubMed http://dx.doi.org/10.1016/j.ijpara.2009.04.003

19. Scallan E, Griffin PM, Angulo FJ, Tauxe RV, Hoekstra RM. Foodborne illness acquired in the United States—unspecified agents. Emerg Infect Dis. 2011;17:16–22. PubMed http://dx.doi.org/10.3201/eid1701.P21101
