# Supplementary material for: Foodborne Illness, Australia, Circa 2000 and Circa 2010
Source: Emerg Infect Dis. 2014 Nov;20(11):1857–64. doi: 10.3201/eid2011.131315 (PMC4214288; doi:10.3201/eid2011.131315)
Supplement: Technical Appendix 3 — Methods used to estimate hospitalizations and deaths due to foodborne illnesses, Australia, circa 2000 and circa 2010. [file 13-1315-Techapp-s3.pdf]

Article DOI: http://dx.doi.org/10.3201/eid2011.131315

Foodborne Illness, Australia, Circa 2000 and Circa 2010

Technical Appendix 3

Methods to Estimate Hospitalizations and Deaths

Data Sources

We used hospitalization data from all Australian States and Territories for 2006–2010 (where available), and deaths data from the Australian Bureau of Statistics, using ICD 10 codes for deaths and ICD 10AM codes for hospitalizations as in Table 1. Both astrovirus and sapovirus were excluded from this analysis as lacking appropriate codes in our data. Patients were included as a hospitalization if the appropriate code was included as the principal or an additional diagnosis. Table 2 shows the percentage of all hospital diagnoses that were listed as the principal diagnosis for each pathogen for 2010 (the year with most complete data). In our previous study (*1*), we used only data on principal diagnoses, with a multiplier of 2 (credible interval [CrI] 1–3) for all pathogens to model both principal and additional diagnoses. It is clear from Table 2 that diagnosis patterns vary considerably by pathogen, so that use of both principal and additional diagnosis data provides a more complete picture of hospitalizations.

Since we only had 1 year of hospitalization data for Victoria and 2 years for New South Wales, we had to extrapolate from these data to the remaining years to derive a distribution of the number of hospitalizations across all states, which was modeled as an empirical distribution. In most cases, we assumed the same number of hospitalizations each year, but some pathogens required further adjustment due to evident outbreaks or trends. For example, an outbreak of hepatitis A associated with sundried tomatoes coincided with the 1 year of hospitalization data for Victoria. We used a ratio of hospitalizations in South Australia to Victoria to estimate Victorian hospitalizations for the missing years. As vaccination against rotavirus resulted in a decrease in incidence, hospitalizations, and deaths, we used data post universal vaccination, from 2008–2010 only, to estimate hospitalizations circa 2010.

Approaches

To calculate estimates of hospitalizations and deaths, we used a statistical model that incorporates uncertainty in case numbers and in multipliers using probability distributions. That is, at each stage of the calculation, the estimate was represented by a probability distribution, and our final estimates and CrIs were computed from this distribution. Figures 1 and 2 provide flowcharts of the approach for hospitalizations, where the left-hand column gives a description of the input or output distribution, the central column provides a representation of the distribution, and the right-hand column describes the type and source of data underlying each input distribution. Input data was obtained from specific data sources (discussed above) or from multipliers that are described below. A fuller description of these probability distributions is provided in the methods section for incidence.

Multipliers

Underdiagnosis Multiplier

Recorded hospitalizations and deaths associated with each pathogen reflect only those individuals that have been tested and confirmed for the pathogen. Following previous studies, we adjusted for this using an underdiagnosis multiplier of 2 (*1*), including a distribution for the multiplier with range 1–3 as in Hall et al. (*2*) and Scallan et al. (*3*). We confirmed the appropriateness of the multiplier for hospitalizations as follows. First, we used the OzFoodNet Outbreak Register to calculate the proportion of all ill cases associated with an outbreak that were hospitalized. We then compared this proportion to the ratio of incidence to hospitalizations both with and without the underdiagnosis multiplier. Although there was some variability by pathogen, overall, we found that 3% of ill cases in the OzFoodNet Outbreak Register were hospitalized. In contrast, the ratio of all incident cases to all hospitalized cases was around 0.01 when the underdiagnosis multiplier was included (and 0.005 otherwise). Although outbreak cases may be more severe than all incident cases (on average), and under-ascertainment of cases or under-recording of hospitalizations may have biased our validation of the multiplier, our results suggest that an underdiagnosis multiplier is appropriate. Further work would assist in better quantifying this multiplier.

Domestically Acquired Multiplier

This multiplier adjusted for the proportion of cases that acquired infection in Australia, and was adopted from the method for incidence. More details of the data and methods behind this multiplier are provided in online Technical Appendix 2 (http://wwwnc.cdc.gov/EID/article/20/11/13-1315-Techapp2.pdf).

Foodborne Multiplier

This multiplier adjusted for the proportion of illness that is foodborne using expert elicitation data, and was used for incidence, hospitalizations and deaths. More details are provided in online Technical Appendix 2.

Hospitalizations and Deaths Due to Unknown Pathogens

A large proportion of hospitalizations and deaths did not identify the source of infection (see “other” codes in Table 1). These data were adjusted and reported as follows for hospitalizations, with a similar approach used for deaths. First, the total number of hospitalizations due to unknown pathogens was calculated from the appropriate codes. We then subtracted from this number the hospitalizations that were attributed to known pathogens according to the underdiagnosis multiplier described above. That is, where total numbers of known gastrointestinal pathogens were increased to adjust for underdiagnosis, this increase was subtracted from the total unknown gastrointestinal pathogens. We assumed a domestically acquired multiplier of 1 for unknown pathogens, but adjusted for the foodborne multiplier using an average over known pathogens, weighted by the number of hospitalizations for each pathogen. For hospitalization data, this gave a foodborne multiplier of 44% (90% CrI 38–50), and for death data, a foodborne multiplier of 51% (90% CrI 36–71). Although Scallan et al. (*3*) do not report their weighted foodborne multipliers for hospitalizations and deaths, analysis of their tables suggest their values are 24% for hospitalizations and 52% for deaths. As noted in online Technical Appendix 2, our calculations are entirely independent; our hospitalization estimate is considerably higher although the estimate for deaths shows good agreement.

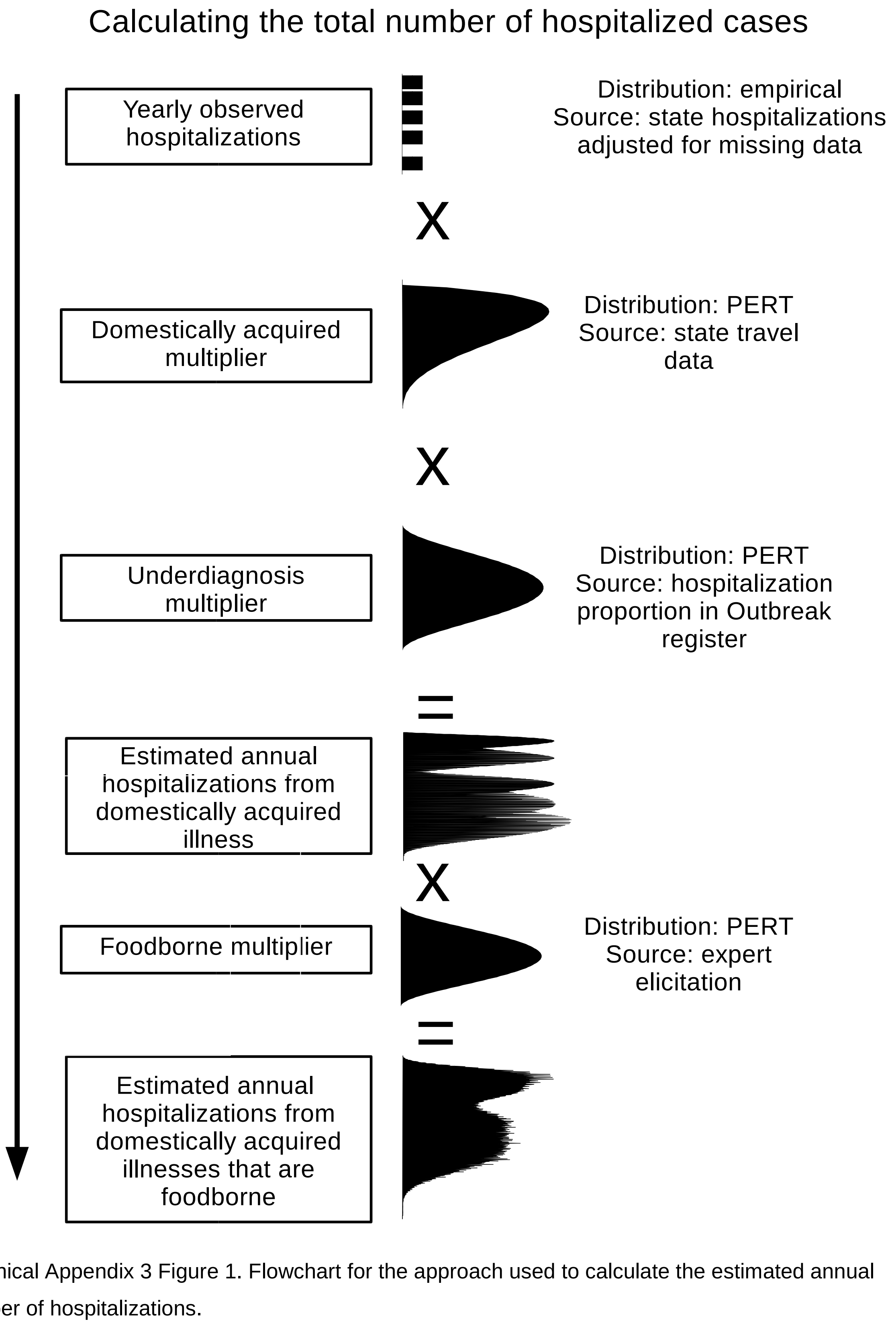

Technical Appendix 3 Figure 1. Flowchart for the approach used to calculate the estimated annual number of hospitalizations.

Calculating the total number of deaths

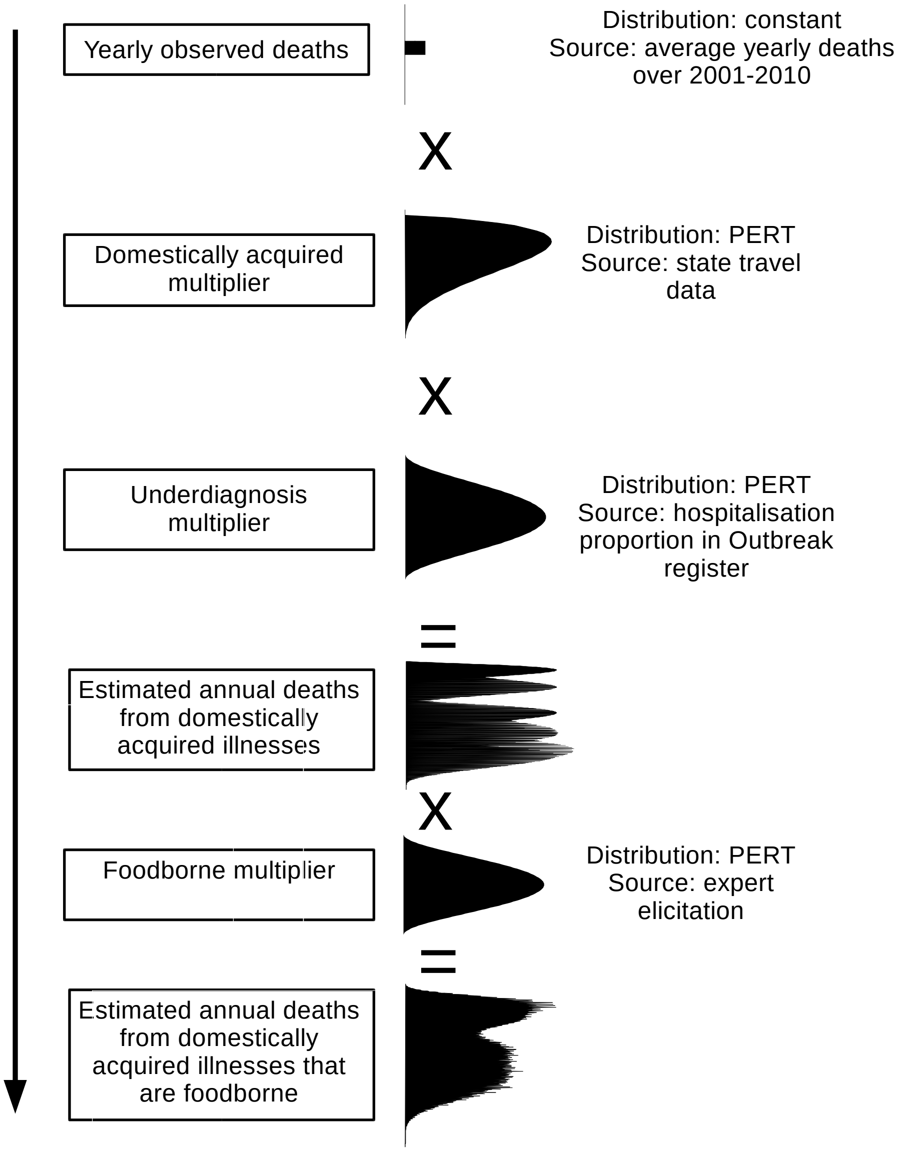

Technical Appendix 3 Figure 2. Flowchart for the approach used to calculate the estimated annual number of deaths.

Technical Appendix 3 Table 1. Mortality and Hospitalization codes for each pathogen*

Pathogen or Illness	Mortality ICD 10 Code and description	ICD 10AM
Adenovirus	A08.2: Adenoviral enteritis	A08.2: Adenoviral enteritis
Bacillus cereus	A05.4: Foodborne *Bacillus cereus* intoxication	A05.4: Foodborne *Bacillus cereus* intoxication
Campylobacter spp.	A04.5: *Campylobacter* enteritis	A04.5: *Campylobacter enteritis*
Ciguatera	T61.0: Ciguatera fish poisoning	T61.0: Ciguatera fish poisoning
Clostridium perfringens	A05.2: Foodborne Clostridium perfringens intoxication	A05.2: Foodborne *Clostridium perfringens* intoxication
Cryptosporidium spp.	A07.2: Cryptosporidiosis	A07.2: Cryptosporidiosis
Guillain-Barré Syndrome	G61.0: Guillain-Barré syndrome	G61.0: Guillain-Barré syndrome
Giardia lamblia	A07.1: Giardiasis [lambliasis]	A07.1: Giardiasis [lambliasis]
Hepatitis A	B15: Acute hepatitis A	B15.9: Hepatitis A without hepatic coma

Pathogen or Illness	Mortality ICD 10 Code and description	ICD 10AM
Hemolytic-uremic syndrome	D59.3: Hemolytic-uremic syndrome	D59.3: Hemolytic-uremic syndrome
Irritable bowel Syndrome	K58: Irritable bowel syndrome	K58.0: Irritable bowel with diarrhea<br><br>K58.9: Irritable bowel without diarrhea
Listeria monocytogenes	A32: Listeriosis	A32.0-A32.9: Listeriosis
Norovirus	A08.1: Acute gastroenteropathy due to Norwalk agent	A08.1: Acute gastroenteropathy due to Norwalk agent
Other pathogenic *Escherichia coli*	A04.0: Enteropathogenic *Escherichia coli* infection<br><br>A04.1: Enterotoxigenic *Escherichia coli* infection<br>A04.2: Enteroinvasive *Escherichia coli* infection<br>A04.4: Other intestinal *Escherichia coli* infection	A04.0: Enteropathogenic *Escherichia coli* infection<br><br>A04.1: Enterotoxigenic *Escherichia coli* infection<br>A04.2: Enteroinvasive *Escherichia coli* infection<br>A04.4: Other intestinal *Escherichia coli* infections
Reactive arthritis	M02.1: Postdysenteric arthropathy<br><br><br>M02.8: Other reactive arthropathies	M02.1: Postdysenteric arthropathy, multiple sites<br>M02.3: Reiter's disease, multiple sites<br>M02.8: Other reactive arthropathies, multiple sites<br>M03.2: Other postinfectious arthropathies in diseases classified elsewhere, multiple sites
Rotavirus	A08.0: Rotaviral enteritis	A08.0: Rotaviral enteritis
Salmonella spp., nontyphoidal†	A02: other *Salmonella* infections	A02.0-A02.9: Salmonellosis
Salmonella enterica serotype Typhi	A01: Typhoid and paratyphoid fevers	A01: Typhoid fever
Scombrotoxicosis	T61.1: Scombroid fish poisoning	T61.6: Scombroid fish poisoning
Shigella spp.	A03: Shigellosis	A03.0-A03.9: Shigellosis
Staphylococcus aureus	A5.0: Foodborne staphylococcal intoxication	A05.0: Foodborne staphylococcal intoxication
STEC	A04.3: Enterohemorrhagic *Escherichia coli* infection	A04.3: Enterohemorrhagic *Escherichia coli* infection
Toxoplasma gondii	B58: Toxoplasmosis	B58.0-B58.9: Toxoplasmosis
Vibrio parahaemolyticus	A05.3: Foodborne *Vibrio parahaemolyticus* intoxication	A05.3: Foodborne *Vibrio parahaemolyticus* intoxication
Yersinia enterocolitica	A04.6: Enteritis due to *Yersinia enterocolitica*	A04.6: Enteritis due to *Yersinia enterocolitica*
Other	A04.8: Other specified bacterial intestinal infection<br>A04.9: Bacterial intestinal infection unspecified<br><br>A05.8: Other specified bacterial foodborne intoxications<br>A05.9: Bacterial foodborne intoxication unspecified<br><br>A07.8: Other specified protozoa intestinal diseases<br>A07.9: Protozoa intestinal disease, unspecified<br>A08.3: Other viral enteritis<br>A08.4: Viral intestinal infection, unspecified<br>A09: Diarrhea and gastroenteritis of presumed infectious origin<br>T61.2 Other fish and shellfish poisoning<br>T61.8 Toxic effect of other seafood<br>T61.9 Toxic effect of unspecified seafood<br>T62: Toxic effect of other noxious substances eaten as food<br>T64: Toxic effect of aflatoxin and other mycotoxin food contaminants	A08.4: Viral intestinal infection, unspecified<br>A09: Diarrhea and gastroenteritis of presumed infectious origin<br>A09.0: Other gastroenteritis and colitis of infectious origin<br>A09.9: Other gastroenteritis and colitis of unspecified origin

*STEC, Shiga toxin–producing *Escherichia coli*.
†Refers to nontyphoidal *Salmonella enterica* serotypes.

Technical Appendix 3 Table 2. The percentage of all hospital diagnoses that were listed as principal for each pathogen, based on 2010 data for all States*

Pathogen or Illness	Percentage of all diagnoses listed as principal
Adenovirus	82
Bacillus cereus	75
Campylobacter spp.	79
Ciguatera	83
Clostridium perfringens	100
Cryptosporidium spp.	59

Pathogen or Illness	Percentage of all diagnoses listed as principal
Other pathogenic *Escherichia coli*	59
Giardia lamblia	34
Guillain-Barré syndrome	71
Irritable bowel syndrome	69
Hemolytic uremic syndrome	30
Hepatitis A	77
Listeria monocytogenes	48
Norovirus	37
Reactive arthritis	50
Rotavirus	77
Salmonella spp., nontyphoidal†	77
Salmonella enterica serotype Typhi	93
Scombrotoxicosis	100
Shigella spp.	76
Staphylococcus aureus	100
STEC	59
Toxoplasma gondii	39
Vibrio parahaemolyticus	50
Yersinia enterocolitica	64

*STEC, Shiga toxin–producing *Escherichia coli.*
†Refers to nontyphoidal *Salmonella enterica* serotypes.

References

1. Hall G, Kirk M, Becker N, Gregory J, Unicomb L, Millard G, et al. Estimating foodborne gastroenteritis, Australia. Emerg Infect Dis. 2005;11:1257–64. PubMed http://dx.doi.org/10.3201/eid1108.041367

2. Mead PS, Slutsker L, Dietz V, McCraig LF, Bresee JS, Shapiro C, et al. Food-related illness and death in the United States. Emerg Infect Dis. 1999;5:607–25. PubMed http://dx.doi.org/10.3201/eid0505.990502

3. Scallan E, Hoekstra RM, Angulo FJ, Tauxe RV, Widdowson MA, Roy SL, et al. Foodborne illness acquired in the United States—major pathogens. Emerg Infect Dis. 2011;17:7–15. PubMed http://dx.doi.org/10.3201/eid1701.P11101
