# Supplementary material for: Foodborne Illness, Australia, Circa 2000 and Circa 2010
Source: Emerg Infect Dis. 2014 Nov;20(11):1857–64. doi: 10.3201/eid2011.131315 (PMC4214288; doi:10.3201/eid2011.131315)
Supplement: Technical Appendix 4 — Pathogen and illness sheets for foodborne illnesses, Australia, circa 2000 and circa 2010. [file 13-1315-Techapp-s4.pdf]

Article DOI: http://dx.doi.org/10.3201/eid2011.131315

Foodborne Illness, Australia, Circa 2000 and Circa 2010

Technical Appendix 4

Pathogen and Illness Sheets

Adenovirus

Technical Appendix 4 Table 1. Primary Data: Water Quality Study; Alternate Data: IID2*

Model Input, Source and Comments	Distribution	Data for Model Input
Reported illness:		
Gastroenteritis multiplier—based on the 2008 National Gastroenteritis Survey	Alternate PERT	2.5%, median, 97.5% values: 0.64, 0.74, 0.84
Pathogen fraction multiplier—based on age adjusted water quality study of an estimated 4 positive isolates per 713 specimens, (Hellard et al. (*1*))	Alternate PERT	2.5%, median, 97.5% values: 0.0015, 0.0056, 0.0143
Population adjustment:<br>Australian resident population 2006–2010 June quarter http://www.abs.gov.au/AUSSTATS/abs@.nsf/DetailsPage/3101.0Dec%202011?OpenDocument (cited 2012 Aug 16)	Empirical	By year (2006–2010): 20697880, 21015936, 21384427, 21778845, 22065317
Domestically acquired multiplier:<br>All illnesses in the Water Quality Study were domestically acquired		NA
Time trend multiplier:<br>No time trend		NA
Underreporting:<br>Water Quality Study is community surveillance		NA
Total illness:<br>Population at risk x gastroenteritis multiplier x pathogen fraction multiplier x time trend multiplier	Outcome	5%, median, 95% values: 28800, 88400, 205000
Rate of total illness per million:<br>Circa 2010	Outcome	5%, median, 95% values: 1300, 4150, 9675
Foodborne multiplier:<br>Assumed to be the same as rotavirus	Alternate PERT	5%, median, 95% values: 0.01, 0.02, 0.03
Total foodborne illness:<br>Total illness x foodborne multiplier	Outcome	5%, median, 95% values: 500, 1650, 4650
Rate of foodborne illness per million:<br>Circa 2010	Outcome	5%, median, 95% values: 25, 80, 215

*Longitudinal study of infectious intestinal disease in the UK. NA, not applicable.

Astrovirus

Technical Appendix 4 Table 2. Primary Data: Water Quality Study; Alternate Data: NA*

Model Input, Source and Comments	Distribution	Data for Model Input
Reported illness:		
Gastroenteritis multiplier—based on the 2008 National Gastroenteritis Survey	Alternate PERT	2.5%, median, 97.5% values: 0.64, 0.74, 0.84
Pathogen fraction multiplier—based on age adjusted water quality study of an estimated 4 positive isolates per 713 specimens, (Hellard et al. (*1*))	Alternate PERT	2.5%, median, 97.5% values: 0.0015, 0.0056, 0.0143
Pathogen comparison multiplier - Kirkwood multiplier (*2*) comparing adenovirus to astrovirus	Constant	0.76
Population adjustment: Australian resident population 2006–2010 June quarter http://www.abs.gov.au/AUSSTATS/abs@.nsf/DetailsPage/3101.0Dec%202011?OpenDocument (cited 2012 Aug 16)	Empirical	By year (2006–2010): 20697880, 21015936, 21384427, 21778845, 22065317
Domestically acquired multiplier: All illnesses in the Water Quality Study were domestically acquired		NA
Time trend multiplier: No time trend		NA
Underreporting: Water Quality Study is community surveillance		NA
Total illness: Population at risk x gastroenteritis multiplier x pathogen fraction multiplier x time trend multiplier	Outcome	5%, median, 95% values: 20900, 67100, 15500
Rate of total illness per million: Circa 2010	Outcome	5%, median, 95% values: 1000, 3150, 7250
Foodborne multiplier: Assumed to be the same as rotavirus	Alternate PERT	5%, median, 95% values: 0.01, 0.02, 0.03
Total foodborne illness: Total illness x foodborne multiplier	Outcome	5%, median, 95% values: 350, 1300, 3400
Rate of foodborne illness per million: Circa 2010	Outcome	5%, median, 95% values: 20, 60, 160

*NA, not applicable.

Bacillus cereus

Technical Appendix 4 Table 3. Primary Data: Outbreak; Alternate Data: NA*

Model Input, Source and Comments	Distribution	Data for Model Input
Reported illness: The number of *B. cereus* outbreak-associated illnesses reported to OzFoodNet 2006–2008	Empirical	By year (2006–2008): 14, 35, 75
Population adjustment: Australian resident population 2006–2010 June quarter http://www.abs.gov.au/AUSSTATS/abs@.nsf/DetailsPage/3101.0Dec%202011?OpenDocument (cited 2012 Aug 16)	Empirical	By year (2006–2008): 20697880, 21015936, 21384427
Domestically acquired multiplier: Assumed to be 100% domestically acquired due to the short incubation period	PERT	Minimum, modal, maximum values: 1, 1, 1
Underreporting:		

Model Input, Source and Comments	Distribution	Data for Model Input
Outbreak multiplier used to adjust from outbreak to surveillance (O-S)<br>Multiplier used to adjust for underreporting from surveillance to community (S-C). Nontyphoidal *Salmonella* multiplier adapted from Hall et al. (*3*)	PERT<br>Log Normal	Minimum, modal, maximum values: 5, 14, 20<br>Mean, standard deviation: 7.44, 2.38
Total illness:<br>Outbreak cases x Underreporting(O-S)(S-C) x Proportion travel-related	Outcome	5%, median, 95% values: 900, 3350, 10100
Rate of total illness per million:<br>Circa 2010	Outcome	5%, median, 95% values: 40, 150, 485
Foodborne multiplier:<br>Based on 2005 expert elicitation	PERT	Minimum, modal, maximum values: 0.98, 1, 1
Total foodborne illness:<br>Total illness x Foodborne multiplier	Outcome	5%, median, 95% values: 2900, 3350, 10100
Rate of foodborne illness per million:<br>Circa 2010	Outcome	5%, median, 95% values: 40, 150, 485

*NA, not applicable.

Campylobacter **spp.**

Technical Appendix 4 Table 4. Primary Data: National Notifiable Disease Surveillance System (NNDSS); Alternate Data: Water Quality Study

Model Input, Source and Comments	Distribution	Data for Model Input
Reported illness:<br>NNDSS data. Available from: http://www9.health.gov.au/cda/source/rpt_4.cfm (Cited 2013 Nov 12)	Empirical	By year (1996–2000): 12169, 11984, 12647, 12373, 13676<br>By year (2006–2010): 15416, 16980, 15539, 16075, 16967
Population adjustment:<br>Australian resident population 2006–2010 June quarter http://www.abs.gov.au/AUSSTATS/abs@.nsf/DetailsPage/3101.0Dec%202011?OpenDocument (cited 2012 Aug 16)	Empirical	By year (1996–2000): 18310714, 18517564, 18711271, 18925855, 19153380<br>By year (2006–2010): 20697880, 21015936, 21384427, 21778845, 22065317
Correction factor:<br>*Campylobacte*r spp. is not notifiable in New South Wales—based on Hall et al (*3*)	Constant	1.5
Domestically acquired multiplier:<br>NNDSS travel data	PERT	Minimum, modal, maximum values: 0.91, 0.97, 0.99
Underreporting:<br>Multiplier used to adjust for underreporting from surveillance to community (S-C). *Campylobacter* spp. multiplier adapted from Hall et al. (*3*)	Log Normal	Mean, standard deviation: 10.45, 2.98
Total illness:<br>Reported cases (NNDSS) x travel adjustment x underreporting (S-C)	Outcome	5%, median, 95% values: 147000, 234000, 374000
Rate of total illness per million:<br>circa 2010	Outcome	5%, median, 95% values: 6850, 10950, 17415
Foodborne multiplier:<br>Expert elicitation study 2009	Alternate PERT	5%, median, 95% values: 0.62, 0.77, 0.89
Total foodborne illness:<br>Total illness x foodborne multiplier	Outcome	5%, median, 95% values: 1108500, 179000, 290000 (circa 2010)<br>5%, median, 95% values: 82500, 139000, 227000 (circa 2000)
Rate of foodborne illness per million:<br>Circa 2010 and circa 2000	Outcome	5%, median, 9% values: 5050, 8400, 13650 (circa 2010)<br>5%, median, 9% values: 4500, 7400, 12200 (circa 2000)

Ciguatera

Technical Appendix 4 Table 5. Primary Data: Queensland Notifications; Alternate Data: Outbreak

Model Input, Source and Comments	Distribution	Data for Model Input
Reported illness:<br>The number of ciguatera notifications reported in Queensland in OzFoodNet Queensland Annual Reports 2006–2010	Empirical	By year (2006–2010): 26, 18, 14, 7, 30
Population adjustment:<br>Australian resident population 2006–2010 June quarter http://www.abs.gov.au/AUSSTATS/abs@.nsf/DetailsPage/3101.0Dec%202011?OpenDocument (cited 2012 Aug 16)	Empirical	By year (2006–2010): 20697880, 21015936, 21384427, 21778845, 22065317
Correction factor:<br>Based on the Queensland and Northern Territory population	Constant	1.05
Domestically acquired multiplier:<br>Assumed to be 100% domestically acquired	PERT	Minimum, modal, maximum values: 1, 1, 1
Underreporting:<br>Multiplier used to adjust for underreporting from surveillance to community (S-C). Nontyphoidal *Salmonella* multiplier adapted from Hall et al (*3*)	Log Normal	Mean, standard deviation: 7.44, 2.38
Total illness:<br>Reported cases (Queensland notifications) x population adjustment x underreporting(O-S)(S-C) x Proportion travel-related	Outcome	5%, median, 95% values: 40, 150, 300
Rate of total illness per million:<br>Circa 2010	Outcome	5%, median, 95% values: 2, 7, 14
Foodborne multiplier:<br>Assumed to be 100% foodborne	PERT	Minimum, modal, maximum values: 1, 1, 1
Total foodborne illness:<br>Total illness x foodborne multiplier	Outcome	5%, median, 95% values: 40, 150, 300
Rate of foodborne illness per million:<br>Circa 2010	Outcome	5%, median, 9% values: 2, 7, 14

Clostridium perfringens

Technical Appendix 4 Table 6. Primary Data: Outbreak; Alternate Data: Water Quality Study

Model Input, Source and Comments	Distribution	Data for Model Input
Reported illness:<br>The number of *C. perfringens* outbreak-associated illnesses reported to OzFoodNet 2006–2008.	Empirical	By year (2006–2008): 183, 44, 383
Population adjustment:<br>Australian resident population 2006–2010 June quarter http://www.abs.gov.au/AUSSTATS/abs@.nsf/DetailsPage/3101.0Dec%202011?OpenDocument (cited 2012 Aug 16)	Empirical	By year (2006–2008): 20697880, 21015936, 21384427
Domestically acquired multiplier:<br>Assumed to be 100% domestically acquired due to the short incubation period	PERT	Minimum, modal, maximum values: 1, 1, 1
Underreporting:<br>Outbreak multiplier used to adjust from outbreak to surveillance (O-S)<br>Multiplier used to adjust for underreporting from surveillance to community (S-C). Nontyphoidal *Salmonella* multiplier adapted from Hall et al. (*3*)	 PERT<br>Log Normal	 Minimum, modal, maximum values: 5, 14, 20<br>Mean, standard deviation: 7.44, 2.38

Model Input, Source and Comments	Distribution	Data for Model Input
Total illness:<br>Outbreak cases x underreporting(O-S)(S-C) x proportion travel-related	Outcome	5%, median, 95% values: 2600, 16500, 53400
Rate of total illness per million:<br>Circa 2010	Outcome	5%, median, 95% values: 35, 785, 2465
Foodborne multiplier:<br>Expert elicitation study 2009	PERT	Minimum, modal, maximum values: 0.86, 0.98, 1
Total foodborne illness:<br>Total illness x foodborne multiplier	Outcome	5%, median, 95% values: 2550, 16100, 50600
Rate of foodborne illness per million:<br>Circa 2010	Outcome	5%, median, 95% values: 130, 765, 2350

Cryptosporidium **spp.**

Technical Appendix 4 Table 7. Primary Data: National Notifiable Disease Surveillance System (NNDSS); Alternate Data: Water Quality Study

Model Input, Source and Comments	Distribution	Data for Model Input
Reported illness:<br>NNDSS data. Available from: http://www9.health.gov.au/cda/source/rpt_4.cfm (cited 2013 Nov 12)	Empirical	By year (2006–2010): 3201, 2809, 2004, 4624, 1479
Population adjustment:<br>Australian resident population 2006–2010 June quarter http://www.abs.gov.au/AUSSTATS/abs@.nsf/DetailsPage/3101.0Dec%202011?OpenDocument (cited 2012 Aug 16)	Empirical	By year (2006–2010): 20697880, 21015936, 21384427, 21778845, 22065317
Domestically acquired multiplier:<br>NNDSS travel data	PERT	Minimum, modal, maximum values: 0.92, 0.97, 0.99
Underreporting:<br>Multiplier used to adjust for underreporting from surveillance to community (S-C). Nontyphoidal *Salmonella* multiplier adapted from Hall et al. (*3*)	Log Normal	Mean, standard deviation: 7.44, 2.38
Total illness:<br>Reported cases (NNDSS) x travel adjustment x underreporting (S-C)	Outcome	5%, median, 95% values: 8150, 17900, 39800
Rate of total illness per million:<br>Circa 2010	Outcome	5%, median, 95% values: 365, 850, 1860
Foodborne multiplier:<br>Based on 2005 expert elicitation	Alternate PERT	5%, median, 95% values: 0.01, 0.1, 0.27
Total foodborne illness:<br>Total illness x foodborne multiplier	Outcome	5%, median, 95% values: 150, 1700, 6100
Rate of foodborne illness per million:<br>Circa 2010	Outcome	5%, median, 9% values: 57, 80, 320

Giardia lamblia

Technical Appendix 4 Table 8. Primary Data: Victoria Notifications; Alternate Data: Water Quality Study

Model Input, Source and Comments	Distribution	Data for Model Input
Reported illness:<br>Victorian State notifications from: O'Grady and Tallis (*4*); Brown et al. (*5–8*). Giardiasis became a non-notifiable disease in Victoria in 2010	Empirical	By year (1996–2000):1085, 1060, 999, 921, 866<br>By year (2006–2009): 1192, 1382, 1434, 1433
Population adjustment:<br>Australian resident population 2006–2010 June quarter http://www.abs.gov.au/AUSSTATS/abs@.nsf/DetailsPage/3101.0Dec%202011?OpenDocument (cited 2012 Aug 16)	Empirical	By year (1996–2000): 18310714, 18517564, 18711271, 18925855, 19153380<br>By year (2006–2009): 20697880, 21015936, 21384427, 21778845
Correction factor:<br>Based on the Victoria population	Constant	4.03
Domestically acquired multiplier:<br>Victorian notification data (*9*)	PERT	Minimum, modal, maximum values: 0.84, 0.85, 0.89
Underreporting:<br>Multiplier used to adjust for underreporting from surveillance to community (S-C). Nontyphoidal *Salmonella* multiplier adapted from Hall et al (*3*)	Log Normal	Mean, standard deviation: 7.44, 2.38
Total illness:<br>Reported cases (Victoria notifications) x population adjustment x underreporting (O-S)(S-C) x proportion travel-related	Outcome	5%, median, 95% values: 19800, 32800, 56400
Rate of total illness per million:<br>Circa 2010	Outcome	5%, median, 95% values: 920, 1560, 2665
Foodborne multiplier:<br>Based on 2005 expert elicitation	PERT	Minimum, modal, maximum values: 0.01, 0.06, 0.5
Total foodborne illness:<br>Total illness x foodborne multiplier	Outcome	5%, median, 95% values: 800, 3700, 10600 (circa 2010)<br>5%, median, 95% values: 565, 2600, 7400 (circa 2000)
Rate of foodborne illness per million:<br>Circa 2010 and circa 2000	Outcome	5%, median, 9% values: 35, 175, 490 (circa 2010)<br>5%, median, 9% values: 30, 140, 405 (circa 2000)

Hepatitis A

Technical Appendix 4 Table 9. Primary Data: National Notifiable Disease Surveillance System (NNDSS); Alternate Data: NA*

Model Input, Source and Comments	Distribution	Data for Model Input
Reported illness:<br>NNDSS data. Available from: http://www9.health.gov.au/cda/source/rpt_4.cfm (cited 2013 Nov 12)	Empirical	By year (1996–2000): 2058, 3032, 2466, 1551, 809<br>By year (2006–2010): 281, 166, 277, 564, 267
Population adjustment:<br>Australian resident population 2006–2010 June quarter http://www.abs.gov.au/AUSSTATS/abs@.nsf/DetailsPage/3101.0Dec%202011?OpenDocument (cited 2012 Aug 16)	Empirical	By year (1996–2000): 18310714, 18517564, 18711271, 18925855, 19153380<br>By year (2006–2010): 20697880, 21015936, 21384427, 21778845, 22065317
Domestically acquired multiplier:<br>NNDSS travel data	PERT	Minimum, modal, maximum values: 0.42, 0.58, 0.77
Underreporting:<br>Multiplier used to adjust for underreporting from surveillance to community (S-C).	Alternate Pert	2.5%, median, 97.5% values: 1, 2, 3

Model Input, Source and Comments	Distribution	Data for Model Input
Total illness:<br>Reported cases (NNDSS) x travel adjustment x underreporting (S-C)	Outcome	5%, median, 95% values: 150, 300, 800
Rate of total illness per million:<br>Circa 2010	Outcome	5%, median, 95% values: 7, 15, 35
Foodborne multiplier:<br>Expert elicitation study 2009	Alternate PERT	5%, median, 95% values: 0.05, 0.12, 0.24
Total foodborne illness:<br>Total illness x foodborne multiplier	Outcome	5%, median, 95% values: 10, 40, 100 (circa 2010)<br>5%, median, 95% values: 65, 245, 725 (circa 2000)
Rate of foodborne illness per million:<br>Circa 2010 and circa 2000	Outcome	5%, median, 9% values: 1, 2, 5 (circa 2010)<br>5%, median, 9% values: 3, 13, 40 (circa 2000)

*NA, not applicable.

Listeria monocytogenes

Technical Appendix 4 Table 10. Primary Data: National Notifiable Disease Surveillance System (NNDSS); Alternate Data: Outbreak

Model Input, Source and Comments	Distribution	Data for Model Input
Reported illness:<br>NNDSS data. Available from: http://www9.health.gov.au/cda/source/rpt_4.cfm (cited 2013 Nov 12)	Empirical	By year (1996–2000): 66, 74, 53, 63, 67<br>By year (2006–2010): 61, 50, 68, 92, 71
Population adjustment:<br>Australian resident population 2006–2010 June quarter http://www.abs.gov.au/AUSSTATS/abs@.nsf/DetailsPage/3101.0Dec%202011?OpenDocument (cited 2012 Aug 16)	Empirical	By year (1996–2000): 18310714, 18517564, 18711271, 18925855, 19153380<br>By year (2006–2010): 20697880, 21015936, 21384427, 21778845, 22065317
Domestically acquired multiplier:<br>Assumed to be 100% because most of the travelers are not at high risk	PERT	Minimum, modal, maximum values: 1, 1, 1
Underreporting:<br>Multiplier used to adjust for underreporting from surveillance to community (S-C).	Alternate Pert	2.5%, median, 97.5% values: 1, 2, 3
Total illness:<br>Reported cases (NNDSS) x travel adjustment x underreporting (S-C)	Outcome	5%, median, 95% values: 50, 150, 200
Rate of total illness per million:<br>circa 2010	Outcome	5%, median, 95% values: 3, 7, 75
Foodborne multiplier:<br>Expert elicitation study 2009	Alternate PERT	5%, median, 95% values: 0.9, 0.98, 1
Total foodborne illness:<br>Total illness x foodborne multiplier	Outcome	5%, median, 95% values: 50, 150, 200 (circa 2010)<br>5%, median, 95% values: 70, 125, 185 (circa 2000)
Rate of foodborne illness per million:<br>Circa 2010 and circa 2000	Outcome	5%, median, 9% values: 3, 7, 75 (circa 2010)<br>5%, median, 9% values: 4, 7, 10 (circa 2000)

Norovirus

Technical Appendix 4 Table 11. Primary Data: Water Quality Study; Alternate Data: Outbreak*

Model Input, Source and Comments	Distribution	Data for Model Input
Reported illness:		
Gastroenteritis multiplier—based on the 2008 National Gastroenteritis Survey	Alternate PERT	2.5%, median, 97.5% values: 0.64, 0.74, 0.84
Pathogen fraction multiplier—based on age adjusted water quality study of an estimated 69 positive isolates per 703 specimens, (Sinclair et al. (*10*))	Alternate PERT	2.5%, median, 97.5% values: 0.0772, 0.0982, 0.1226
Population adjustment: Australian resident population 2006–2010 June quarter http://www.abs.gov.au/AUSSTATS/abs@.nsf/DetailsPage/3101.0Dec%202011?OpenDocument (cited 2012 Aug 16)	Empirical	By year (2006–2010): 20697880, 21015936, 21384427, 21778845, 22065317
Domestically acquired multiplier: All illnesses in the Water Quality Study were domestically acquired		NA
Time trend multiplier: No time trend		NA
Underreporting: Water Quality Study is community surveillance		NA
Total illness: Population at risk x gastroenteritis multiplier x pathogen fraction multiplier x time trend multiplier	Outcome	5%, median, 95% values: 1220000, 1550000, 1940000
Rate of total illness per million: Circa 2010	Outcome	5%, median, 95% values: 57100, 72500, 90550
Foodborne multiplier: Expert elicitation study 2009	Alternate PERT	5%, median, 95% values: 0.05, 0.18, 0.35
Total foodborne illness: Total illness x foodborne multiplier	Outcome	5%, median, 95% values: 78100, 276000, 563000
Rate of foodborne illness per million: Circa 2010	Outcome	5%, median, 95% values: 3620, 12920, 26300

*NA, not applicable.

Other pathogenic *Escherichia coli*

Technical Appendix 4 Table 11. Primary Data: Water Quality Study; Alternate Data: IID2*

Model Input, Source and Comments	Distribution	Data for Model Input
Reported illness:		
Gastroenteritis multiplier—based on the 2008 National Gastroenteritis Survey	Alternate PERT	2.5%, median, 97.5% values: 0.64, 0.74, 0.84
Pathogen fraction multiplier—based on age adjusted water quality study of an estimated 50 positive isolates per 713 specimens, (Hellard et al [*1*])	Alternate PERT	2.5%, median, 97.5% values: 0.0525, 0.074, 0.0914
Population adjustment: Australian resident population 2006–2010 June quarter http://www.abs.gov.au/AUSSTATS/abs@.nsf/DetailsPage/3101.0Dec%202011?OpenDocument (cited 2012 Aug 16)	Empirical	By year (2006–2010): 20697880, 21015936, 21384427, 21778845, 22065317
Domestically acquired multiplier: All illnesses in the Water Quality Study were domestically acquired		NA

Model Input, Source and Comments	Distribution	Data for Model Input
Time trend multiplier:<br>No time trend		NA
Underreporting:<br>Water Quality Study is community surveillance		NA
Total illness:<br>Population at risk x gastroenteritis multiplier x pathogen fraction multiplier x time trend multiplier	Outcome	5%, median, 95% values: 833000, 1100000, 1450000
Rate of total illness per million:<br>Circa 2010	Outcome	5%, median, 95% values: 39150, 51350, 67550
Foodborne multiplier:<br>Expert elicitation study 2009	Alternate PERT	5%, median, 95% values: 0.08, 0.23, 0.55
Total foodborne illness:<br>Total illness x foodborne multiplier	Outcome	5%, median, 95% values: 85800, 255000, 632000
Rate of foodborne illness per million:<br>Circa 2010	Outcome	5%, median, 95% values: 4100, 11600, 29700

*Longitudinal study of infectious intestinal disease in the UK. NA, not applicable.

Rotavirus

Technical Appendix 4 Table 11. Primary Data: Water Quality Study; Alternate Data: IID2*

Model Input, Source and Comments	Distribution	Data for Model Input
Reported illness:<br>Gastroenteritis multiplier—based on the 2008 National Gastroenteritis Survey	Alternate PERT	2.5%, median, 97.5% values: 0.64, 0.74, 0.84
Pathogen fraction multiplier—based on age adjusted water quality study of an estimated 50 positive isolates per 713 specimens, (Hellard et al. [*1*])	Alternate PERT	2.5%, median, 97.5% values: 0.0031, 0.0084, 0.0182
Population adjustment:<br>Australian resident population 2006–2010 June quarter<br>http://www.abs.gov.au/AUSSTATS/abs@.nsf/DetailsPage/3101.0Dec%202011?OpenDocument (cited 2012 Aug 16)	Empirical	By year (2006–2010):<br>20697880, 21015936, 21384427, 21778845, 22065317
Domestically acquired multiplier:<br>All illnesses in the Water Quality Study were domestically acquired		NA
Time trend multiplier:<br>Based on Dey et al. (*11*)	Alternate PERT	2.5%, median, 97.5% values: 0.318, 0.338, 0.359
Underreporting:<br>Water Quality Study is community surveillance		NA
Total illness:<br>Population at risk x gastroenteritis multiplier x pathogen fraction multiplier x time trend multiplier	Outcome	5%, median, 95% values: 18500, 44800, 90800
Rate of total illness per million:<br>Circa 2010	Outcome	5%, median, 95% values: 875, 2100, 4260
Foodborne multiplier:<br>Expert elicitation study 2009	Alternate PERT	5%, median, 95% values: 0.01, 0.02, 0.03
Total foodborne illness:<br>Total illness x foodborne multiplier	Outcome	5%, median, 95% values: 300, 850, 2000

Model Input, Source and Comments	Distribution	Data for Model Input
Rate of foodborne illness per million:<br>Circa 2010	Outcome	5%, median, 95% values: 15, 40, 95

*Longitudinal study of infectious intestinal disease in the UK. NA, not applicable.

Salmonella spp., nontyphoidal (refers to nontyphoidal *Salmonella enterica* serotypes)

Technical Appendix 4 Table 14. Primary Data: National Notifiable Disease Surveillance System (NNDSS); Alternate Data: Water Quality Study

Model Input, Source and Comments	Distribution	Data for Model Input
Reported illness:<br>NNDSS data. Available from: http://www9.health.gov.au/cda/source/rpt_4.cfm (cited 2013 Nov 12)	Empirical	By year (1996–2000): 5744, 6955, 7513, 7008, 6187<br>By year (2006–2010): 8241, 9502, 8316, 9524, 11928
Population adjustment:<br>Australian resident population 2006–2010 June quarter<br>http://www.abs.gov.au/AUSSTATS/abs@.nsf/DetailsPage/3101.0Dec%202011?OpenDocument (cited 2012 Aug 16)	Empirical	By year (1996–2000): 18310714, 18517564, 18711271, 18925855, 19153380<br>By year (2006–2010): 20697880, 21015936, 21384427, 21778845, 22065317
Domestically acquired multiplier:<br>NNDSS travel data	PERT	Minimum, modal, maximum values: 0.7, 0.85, 0.95
Underreporting:<br>Multiplier used to adjust for underreporting from surveillance to community (S-C)	Log Normal	Mean, standard deviation: 7.44, 2.38
Total illness:<br>Reported cases (NNDSS) x travel adjustment x underreporting(S-C)	Outcome	5%, median, 95% values: 31900, 56200, 101000
Rate of total illness per million:<br>Circa 2010	Outcome	5%, median, 95% values: 1515, 2650, 4650
Foodborne multiplier:<br>Expert elicitation study 2009	Alternate<br>PERT	5%, median, 95% values: 0.53, 0.72, 0.86
Total foodborne illness:<br>Total illness x foodborne multiplier	Outcome	5%, median, 95% values: 21200, 39600, 73400 (circa 2010)<br>5%, median, 95% values: 15000, 28000, 50000 (circa 2000)
Rate of foodborne illness per million:<br>Circa 2010 and circa 2000	Outcome	5%, median, 9% values: 1000, 1850, 3350 (circa 2010)<br>5%, median, 9% values: 800, 1500, 2700 (circa 2000)

Salmonella enterica serotype Typhi

Technical Appendix 4 Table 15. Primary Data: National Notifiable Disease Surveillance System (NNDSS); Alternate Data: NA*

Model Input, Source and Comments	Distribution	Data for Model Input
Reported illness:<br>NNDSS data. Available from: http://www9.health.gov.au/cda/source/rpt_4.cfm (cited 2013 Nov 12)	Empirical	By year (1996–2000): 72, 72, 57, 63, 58<br>By year (2006–2010): 77, 90, 105, 115, 95
Population adjustment:<br>Australian resident population 2006–2010 June quarter<br>http://www.abs.gov.au/AUSSTATS/abs@.nsf/DetailsPage/3101.0Dec%202011?OpenDocument (cited 2012 Aug 16)	Empirical	By year (1996–2000): 18310714, 18517564, 18711271, 18925855, 19153380<br>By year (2006–2010): 20697880, 21015936, 21384427, 21778845, 22065317
Domestically acquired multiplier:<br>NNDSS travel data	PERT	Minimum, modal, maximum values: 0.02, 0.11, 0.25
Underreporting:	Alternate	2.5%, median, 97.5% values:1, 2, 3

Model Input, Source and Comments	Distribution	Data for Model Input
Multiplier used to adjust for underreporting from surveillance to community (S-C)	PERT	
Total illness:<br>Reported cases (NNDSS) x travel adjustment x underreporting (S-C)	Outcome	5%, median, 95% values: 8, 20, 45
Rate of total illness per million:<br>Circa 2010	Outcome	5%, median, 95% values: 0, 1, 2
Foodborne multiplier:<br>Based on 2005 expert elicitation	PERT	Minimum, modal, maximum values: 0.02, 0.75, 0.97
Total foodborne illness:<br>Total illness x foodborne multiplier	Outcome	5%, median, 95% values: 5, 15, 30 (circa 2010)<br>5%, median, 95% values: 3, 9, 21 (circa 2000)
Rate of foodborne illness per million:<br>Circa 2010 and circa 2000	Outcome	5%, median, 9% values: 0, 0.6, 1 (circa 2010)<br>5%, median, 9% values: 0, 0.5, 1 (circa 2000)

*NA, not applicable.

Sapovirus

Technical Appendix 4 Table 16. Primary Data: Water Quality Study; Alternate Data: IID2*

Model Input, Source and Comments	Distribution	Data for Model Input
Reported illness:<br>Gastroenteritis multiplier—based on the 2008 National Gastroenteritis Survey	Alternate PERT	2.5%, median, 97.5% values: 0.64, 0.74, 0.84
Pathogen fraction multiplier—based on age adjusted water quality study findings for norovirus of an estimated 69 positive isolates per 703 specimens (Sinclair et al. [*10*])	Alternate PERT	2.5%, median, 97.5% values: 0.0772, 0.0982, 0.1226
Pathogen comparison multiplier – Kirkwood multiplier (*2*) comparing norovirus to sapovirus	Constant	0.5
Population adjustment:<br>Australian resident population 2006–2010 June quarter<br>http://www.abs.gov.au/AUSSTATS/abs@.nsf/DetailsPage/3101.0Dec%202011?OpenDocument (cited 2012 Aug 16)	Empirical	By year (2006–2010):<br>20697880, 21015936, 21384427, 21778845, 22065317
Domestically acquired multiplier:<br>All illnesses in the Water Quality Study were domestically acquired		NA
Time trend multiplier:<br>No time trend		NA
Underreporting:<br>Water Quality Study is community surveillance		NA
Total illness:<br>Population at risk x gastroenteritis multiplier x pathogen fraction multiplier x time trend multiplier	Outcome	5%, median, 95% values: 63400, 81600, 102000
Rate of total illness per million:<br>Circa 2010	Outcome	5%, median, 95% values: 3000, 3800, 4800
Foodborne multiplier:<br>Assumed to be the same as norovirus	PERT	Minimum, modal, maximum values: 0.05, 0.18, 0.35
Total foodborne illness:<br>Total illness x foodborne multiplier	Outcome	5%, median, 95% values: 7450, 15000, 24300
Rate of foodborne illness per million:<br>Circa 2010	Outcome	5%, median, 95% values: 350, 700, 1150

*Longitudinal study of infectious intestinal disease in the UK. NA, not applicable.

Scombrotoxicosis

Technical Appendix 4 Table 17. Primary Data: Outbreak; Alternate Data: NA*

Model Input, Source and Comments	Distribution	Data for Model Input
Reported illness:<br>The number of scombrotoxicosis outbreak-associated illnesses reported to OzFoodNet 2006–2008.	Empirical	By year (2006–2008): 12, 17, 0
Population adjustment:<br>Australian resident population 2006–2010 June quarter<br>http://www.abs.gov.au/AUSSTATS/abs@.nsf/DetailsPage/3101.0Dec%202011?OpenDocument<br>(cited 2012 Aug 16)	Empirical	By year (2006–2008): 20697880, 21015936, 21384427
Domestically acquired multiplier:<br>Assumed to be 100% domestically acquired due to the short incubation period	PERT	Minimum, modal, maximum values: 1, 1, 1
Underreporting:<br>Outbreak multiplier used to adjust from outbreak to surveillance (O-S)<br>Multiplier used to adjust for underreporting from surveillance to community (S-C). Nontyphoidal *Salmonella* multiplier adapted from Hall et al (*3*)	 PERT<br>Log Normal	 Minimum, modal, maximum values: 5, 14, 20<br>Mean, standard deviation: 7.44, 2.38
Total Illness:<br>Outbreak cases x underreporting (O-S)(S-C) x proportion travel-related	Outcome	5%, median, 95% values: 0, 1050, 2450
Rate of total illness per million:<br>Circa 2010	Outcome	5%, median, 95% values: 0, 50, 115
Foodborne multiplier:<br>Assumed to be 100% foodborne	PERT	Minimum, modal, maximum values: 1, 1, 1
Total foodborne illness:<br>Total illness x foodborne multiplier	Outcome	5%, median, 95% values: 0, 1050, 2450
Rate of foodborne illness per million:<br>Circa 2010	Outcome	5%, median, 95% values: 0, 50, 115

*NA, not applicable.

Shigella spp.

Technical Appendix 4 Table 17. Primary Data: National Notifiable Disease Surveillance System (NNDSS); Alternate Data: NA*

Model Input, Source and Comments	Distribution	Data for Model Input
Reported illness:<br>NNDSS data. Available from: http://www9.health.gov.au/cda/source/rpt_4.cfm (cited 2013 Nov 12)	Empirical	By year (1996–2000): 660, 802, 580, 534, 488<br>By year (2006–2010): 545, 597, 828, 618, 550
Population adjustment:<br>Australian resident population 2006–2010 June quarter<br>http://www.abs.gov.au/AUSSTATS/abs@.nsf/DetailsPage/3101.0Dec%202011?OpenDocument<br>(cited 2012 Aug 16)	Empirical	By year (1996–2000): 18310714, 18517564, 18711271, 18925855, 19153380<br>By year (2006–2010): 20697880, 21015936, 21384427, 21778845, 22065317
Domestically acquired multiplier:<br>NNDSS travel data	PERT	Minimum, modal, maximum values: 0.45, 0.7, 0.84
Underreporting:<br>Multiplier used to adjust for underreporting from surveillance to community (S-C). Nontyphoidal *Salmonella* spp. multiplier adapted from Hall et al. (*3*)	Log Normal	Mean, standard deviation: 7.44, 2.38
Total Illness:<br>Reported cases (NNDSS) x travel adjustment x underreporting (S-C)	Outcome	5%, median, 95% values: 1650, 3000, 5400
Rate of total illness per million:	Outcome	5%, median, 95% values: 75, 140, 260

Model Input, Source and Comments	Distribution	Data for Model Input
Circa 2010		
Foodborne multiplier:<br>Expert elicitation study 2009	Alternate PERT	5%, median, 95% values: 0.05, 0.12, 0.23
Total foodborne illness:<br>Total illness x foodborne multiplier	Outcome	5%, median, 95% values: 150, 350, 850 (circa 2010)<br>5%, median, 95% values: 175, 515, 1300 (circa 2000)
Rate of foodborne illness per million:<br>Circa 2010 and circa 2000	Outcome	5%, median, 9% values: 6, 16, 40 (circa 2010)<br>5%, median, 9% values: 9, 28, 70 (circa 2000)

*NA, not applicable.

Staphylococcus aureus

Technical Appendix 4 Table 19. Primary Data: Outbreak; Alternate Data: NA*

Model Input, Source and Comments	Distribution	Data for Model Input
Reported illness:<br>The number of *S. aureus* outbreak-associated illnesses reported to OzFoodNet 2006–2008	Empirical	By year (2006–2008): 3, 14, 50
Population adjustment:<br>Australian resident population 2006–2010 June quarter<br>http://www.abs.gov.au/AUSSTATS/abs@.nsf/DetailsPage/3101.0Dec%202011?OpenDocument (cited 2012 Aug 16)	Empirical	By year (2006–2008): 20697880, 21015936, 21384427
Domestically acquired multiplier:<br>Assumed to be 100% domestically acquired due to the short incubation period	PERT	Minimum, modal, maximum values: 1, 1, 1
Underreporting:		
Outbreak multiplier used to adjust from outbreak to surveillance (O-S)	PERT	Minimum, modal, maximum values: 5, 14, 20
Multiplier used to adjust for underreporting from surveillance to community (S-C). Nontyphoidal *Salmonella* multiplier adapted from Hall et al. (*3*)	Log Normal	Mean, standard deviation: 7.44, 2.38
Total Illness:<br>Outbreak cases x underreporting (O-S)(S-C) x proportion travel-related	Outcome	5%, median, 95% values: 200, 1300, 7050
Rate of total illness per million:<br>Circa 2010	Outcome	5%, median, 95% values: 9, 60, 350
Foodborne multiplier:<br>Based on 2005 expert elicitation	PERT	Minimum, modal, maximum values: 0.95, 1, 1
Total foodborne illness:<br>Total illness x foodborne multiplier	Outcome	5%, median, 95% values: 200, 1300, 7000
Rate of foodborne illness per million:<br>Circa 2010	Outcome	5%, median, 95% values: 9, 60, 350

*NA, not applicable.

Shiga toxin–producing *Escherichia coli*

Technical Appendix 4 Table 20. Primary Data: South Australian Surveillance; Alternate Data: National Notifiable Disease Surveillance System

Model Input, Source and Comments	Distribution	Data for Model Input
Reported illness:<br>South Australian State STEC surveillance from the study by Vally et al. (*12*)	Empirical	By year (2006–2010): 35, 40, 39, 62, 32
Population adjustment:<br>Australian resident population 2006–2010 June quarter<br>http://www.abs.gov.au/AUSSTATS/abs@.nsf/DetailsPage/3101.0Dec%202011?OpenDocument<br>(cited 2012 Aug 16)	Empirical	By year (2006–2010):<br>20697880, 21015936, 21384427, 21778845, 22065317
Correction factor:<br>Based on the South Australian population	Constant	13.4
Domestically acquired multiplier:<br>NNDSS travel data	PERT	Minimum, modal, maximum values: 0.93, 0.99, 1
Underreporting:<br>Multiplier used to adjust for underreporting from surveillance to community (S-C). STEC multiplier adapted from Hall et al (*3*)	Log Normal	Mean, standard deviation: 8.83, 3.7
Total illness:<br>Reported cases(SA surveillance) x correction factor x travel adjustment x underreporting (S-C)	Outcome	5%, median, 95% values: 2050, 4300, 9500
Rate of total illness per million:<br>Circa 2010	Outcome	5%, median, 95% values: 100, 200, 450
Foodborne multiplier:<br>Expert elicitation study 2009	Alternate PERT	5%, median, 95% values: 0.32, 0.56, 0.83
Total foodborne illness:<br>Total illness x foodborne multiplier	Outcome	5%, median, 95% values: 950, 2350, 5850
Rate of foodborne illness per million:<br>Circa 2010	Outcome	5%, median, 9% values: 45, 110, 260

Toxoplasma gondii

Technical Appendix 4 Table 21. Primary Data: State and Territory Notifications; Alternate Data: NA*

Model Input, Source and Comments	Distribution	Data for Model Input
Reported illness:<br>US seroprevalence data (*13*) extrapolated to the Australian population for 2010 by age group	Empirical	0-4: 5709<br>5-9: 5749<br>10-19: 10744<br>20-29: 11728<br>30-39: 10809<br>40-49:10377<br>50-59: 8903<br>60-69: 6521<br>70-79:3713<br>80+: 2342<br>Total: 76095
Population adjustment:<br>Australian resident population 2010 by age group June quarter<br>http://www.abs.gov.au/AUSSTATS/abs@.nsf/DetailsPage/3101.0Dec%202011?OpenDocument	Empirical	0-4: 1441679<br>5-9: 1352211<br>10-19: 2852050

Model Input, Source and Comments	Distribution	Data for Model Input
(cited 2012 Aug 16)		20-29: 3240347<br>30-39:3108224<br>40-49: 3105877<br>50-59: 2773511<br>60-69: 2114158<br>70-79: 1253114<br>80+: 824146
Domestically acquired multiplier:<br>Assumed to be 100% domestically acquired	PERT	Minimum, modal, maximum values: 1, 1, 1
Proportion symptomatic:<br>Scallan et al. (*14*) and Abelson et al. (*15*)	PERT	Minimum, modal, maximum values: 0.11, 0.15, 0.21
Total illness:<br>Estimated yearly cases x travel adjustment x proportion symptomatic	Outcome	5%, median, 95% values: 8350, 11400, 16000
Rate of total illness per million:<br>Circa 2010	Outcome	5%, median, 95% values: 380, 515, 760
Foodborne multiplier:<br>Based on 2005 expert elicitation	PERT	Minimum, modal, maximum values: 0.04, 0.31, 0.74
Total foodborne illness:<br>Total illness x foodborne multiplier	Outcome	5%, median, 95% values: 1400, 3750, 7150
Rate of foodborne illness per million:<br>Circa 2010	Outcome	5%, median, 9% values: 65, 170, 325

*NA, not applicable.

Vibrio parahaemolyticus

Technical Appendix 4 Table 22. Primary Data: Western Australia Notifications; Alternate Data: NA*

Model Input, Source and Comments	Distribution	Data for Model Input
Reported illness:<br>Western Australia Notifications—<br>http://www.public.health.wa.gov.au/cproot/4195/2/12172_DiseaseWatch.pdf	Empirical	By year (2006–2010): 3, 9, 7, 9, 10
Population adjustment:<br>Australian resident population 2006–2010 June quarter<br>http://www.abs.gov.au/AUSSTATS/abs@.nsf/DetailsPage/3101.0Dec%202011?OpenDocument<br>(cited 2012 Aug 16)	Empirical	By year (2006–2010): 20697880, 21015936, 21384427, 21778845, 22065317
Correction factor:<br>Based on the Western Australia population	Constant	9.61
Domestically acquired multiplier:<br>OzFoodNet WA Annual Reports 2006–2010	PERT	Minimum, modal, maximum values: 0, 0.18, 0.33
Underreporting:<br>Multiplier used to adjust for underreporting from surveillance to community (S-C). Nontyphoidal *Salmonella* multiplier adapted from Hall et al. (*3*)	Log Normal	Mean, standard deviation: 7.44, 2.38
Total Illness:<br>Reported cases (Western Australia notifications) x population adjustment x underreporting (O-S)(S-C) x proportion travel-related	Outcome	5%, median, 95% values: 15, 60, 170
Rate of total illness per million:<br>Circa 2010	Outcome	5%, median, 95% values: 1, 3, 8

Model Input, Source and Comments	Distribution	Data for Model Input
Foodborne multiplier:<br>Based on 2005 expert elicitation	PERT	Minimum, modal, maximum values: 0.05, 0.75, 0.96
Total foodborne illness:<br>Total illness x foodborne multiplier	Outcome	5%, median, 95% values: 10, 40, 120
Rate of foodborne illness per million:<br>Circa 2010	Outcome	5%, median, 9% values: 0, 2, 6

*NA, not applicable.

Yersinia enterocolitica

Technical Appendix 4 Table 23. Primary Data: State and Territory Notifications; Alternate Data: NA*

Model Input, Source and Comments	Distribution	Data for Model Input
Reported illness:<br>State notifications from Queensland, South Australia, Western Australia, and Northern Territory extrapolated from State data to the Australian population to determine the expected number of notifications if all States were reporting	Empirical	By year (2006–2010): 214, 249, 326, 242, 239
Population adjustment:<br><br>Australian resident population 2006–2010 June quarter http://www.abs.gov.au/AUSSTATS/abs@.nsf/DetailsPage/3101.0Dec%202011?OpenDocument (cited 2012 Aug 16)	Empirical	By year (2006–2010): 20697880, 21015936, 21384427, 21778845, 22065317
Correction factor:<br>Based on the Western Australia population	Constant	9.61
Domestically acquired multiplier:<br>OzFoodNet Western Australia Annual Reports 2006–2010	PERT	Minimum, modal, maximum values: 0.8, 0.9, 1
Underreporting:<br>Multiplier used to adjust for underreporting from surveillance to community (S-C). Nontyphoidal *Salmonella* multiplier adapted from Hall et al (*3*)	Log Normal	Mean, standard deviation: 7.44, 2.38
Total Illness:<br>Reported cases (extrapolated State notifications) x population adjustment x underreporting (O-S)(S-C) x proportion travel-related	Outcome	5%, median, 95% values: 1900, 1500, 2500
Rate of total illness per million:<br>Circa 2010	Outcome	5%, median, 95% values: 140, 70, 115
Foodborne multiplier:<br>Based on 2005 expert elicitation	PERT	Minimum, modal, maximum values: 0.28, 0.84, 0.94
Total foodborne illness:<br>Total illness x foodborne multiplier	Outcome	5%, median, 95% values: 650, 1150, 1950
Rate of foodborne illness per million:<br>Circa 2010	Outcome	5%, median, 9% values: 30, 50, 90

*NA, not applicable.

References

1. Hellard ME, Sinclair MI, Forbes A, Fairley CK. A randomized, blinded, controlled trial investigating the gastrointestinal health effects of drinking water quality. Environ Health Perspect. 2001;109:773–8. PubMed http://dx.doi.org/10.1289/ehp.01109773

2. Kirkwood CD, Clark R, Bogdanovic-Sakran N, Bishop RF. A 5-year study of the prevalence and genetic diversity of human caliciviurses associated with sporadic cases of acute gastroenteritis in young children admitted to hospital in Melbourne, Australia (1998–2002). J Med Virol. 2005;77:96–101. PubMed http://dx.doi.org/10.1002/jmv.20419

3. Hall G, Yohannes K, Raupach J, Becker N, Kirk M. Estimating community incidence of *Salmonella*, *Campylobacter* and Shiga toxin–producing *Escherichia coli* infections, Australia. Emerg Infect Dis. 2008;14:1601–9. PubMed http://dx.doi.org/10.3201/eid1410.071042

4. O'Grady KA, Tallis G, editors. Surveillance of notifiable infectious diseases in Victoria 2000 [cited 2012 Dec 18]. http://docs.health.vic.gov.au/docs/doc/Surveillance-of-notifiable-infectious-diseases-in-Victoria-2000

5. Brown L, Fielding J, Gregory J, Yohannes K, Higgins N, Klug G, et al. Surveillance of notifiable infectious diseases in Victoria, 2006. In: Public Health Branch, editor. Melbourne (Australia): Communicable Disease Control Unit Rural and Regional Health and Aged Care Services, Department of Human Services, Victoria; 2008.

6. Brown L, El-Hayek C, Fielding J, Gregory J, Higgins N, Klug G, et al. Surveillance of notifiable infectious diseases in Victoria, 2007. In: Health Protection Branch, editor. Melbourne (Australia): The Communicable Disease Prevention and Control Unit, Wellbeing, Integrated Care and Ageing, Department of Human Services, Victoria; 2010.

7. Brown L, El-Hayek C, Franklin L, Gregory J, Higgins N, Klug G, et al. Surveillance of notifiable infectious diseases in Victoria, 2008. In: Health Protection Branch, editor. Melbourne (Australia): The Communicable Disease Prevention and Control Unit, Wellbeing, Integrated Care and Ageing, Department of Human Services, Victoria; 2011.

8. Brown L, El-Hayek C, Franklin L, Gregory J, Higgins N, Klug G, et al. Surveillance of notifiable infectious diseases in Victoria, 2009. In: Health Protection Branch, editor Melbourne (Australia): Communicable Disease Control Unit Rural and Regional Health and Aged Care Services, Department of Human Services, Victoria; 2011.

9. Williams S. Personal communication. 2013.

10. Sinclair MI, Hellard ME, Wolfe R, Mitakakis TZ, Leder K, Fairley CK. Pathogens causing community gastroenteritis in Australia. J Gastroenterol Hepatol. 2005;20:1685–90 . PubMed http://dx.doi.org/10.1111/j.1440-1746.2005.04047.x

11. Dey A, Wang H, Menzies R, Macartney K. Changes in hospitalisations for acute gastroenteritis in Australia after the national rotavirus vaccination program. Med J Aust. 2012;197:453–7. PubMed http://dx.doi.org/10.5694/mja12.10062

12. Vally H, Hall G, Dyda A, Raupach J, Knope K, Combs B, et al. Epidemiology of Shiga toxin producing *Escherichia coli* in Australia, 2000–2010. BMC Public Health. 2012;12:63–74. PubMed http://dx.doi.org/10.1186/1471-2458-12-63

13. Jones JL, Kruszon-Moran D, Sanders-Lewis K, Wilson M. *Toxoplasma gondii* infection in the United States, 1999–2004, decline from the prior decade. Am J Trop Med Hyg. 2007;77:405–10. PubMed

14. Scallan E, Hoekstra RM, Angulo FJ, Tauxe RV, Widdowson MA, Roy SL, et al. Foodborne illness acquired in the United States—major pathogens. Emerg Infect Dis. 2011;17:7–15. PubMed http://dx.doi.org/10.3201/eid1701.P11101

15. Abelson P, Potter Forbes M, Hall G. The annual cost of foodborne illness in Australia. Canberra (Australia): Commonwealth Department of Health and Ageing; March 2006.
