# Supplementary material for: Foodborne Illness, Australia, Circa 2000 and Circa 2010
Source: Emerg Infect Dis. 2014 Nov;20(11):1857–64. doi: 10.3201/eid2011.131315 (PMC4214288; doi:10.3201/eid2011.131315)
Supplement: Technical Appendix 5 — Median numbers of gastroenteritis and nongastrointestinal illness cases caused by all domestically acquired pathogens and by domestically acquired foodborne pathogens, Australia, circa 2010. [file 13-1315-Techapp-s5.pdf]

Article DOI: http://dx.doi.org/10.3201/eid2011.131315

Foodborne Illness, Australia, Circa 2000 and Circa 2010

Technical Appendix 5

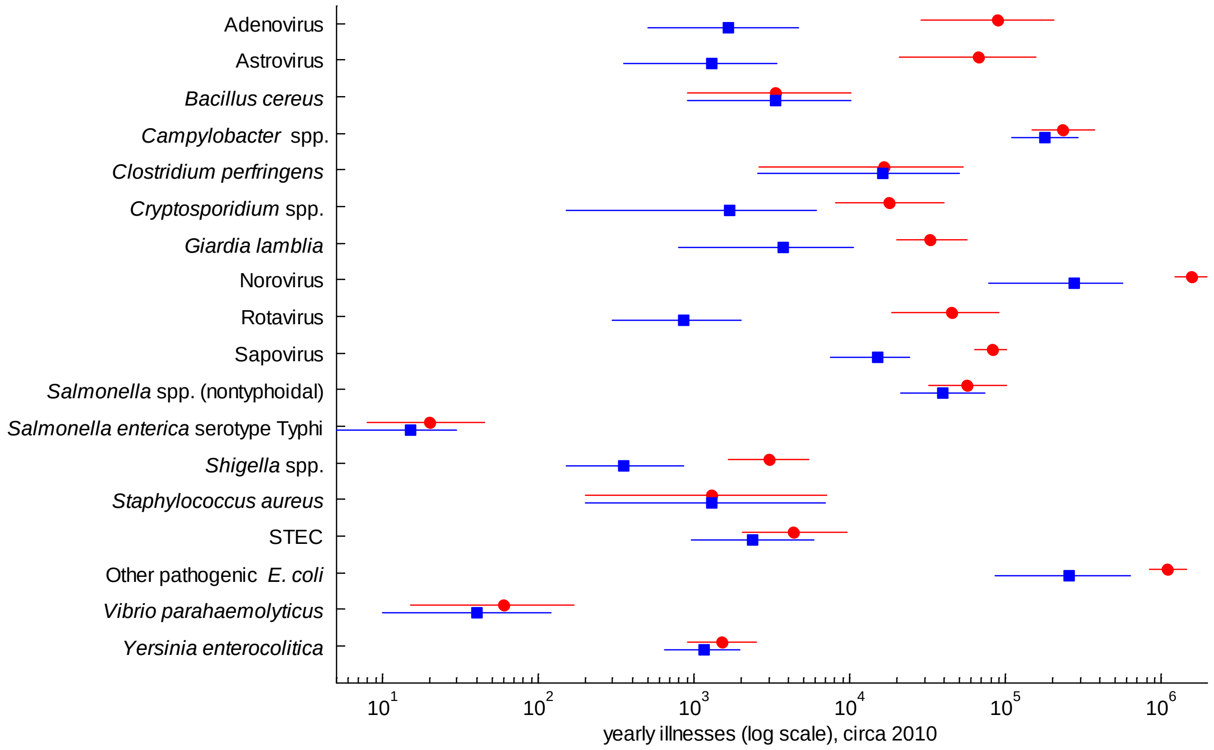

Technical Appendix 5 Figure. Median number of all domestically acquired illnesses (red dots) and domestically acquired foodborne illnesses (blue squares), by pathogen, Australia, circa 2010. Bars indicate 90% credible intervals. *E. coli*, *Escherichia coli*; STEC, Shiga toxin–producing *E. coli*. *Salmonella* spp. (nontyphoidal) refers to nontyphoidal *Salmonella enterica* serotypes.
